# A Systematic Evaluation of Short Tandem Repeats in Lipid Candidate Genes: Riding on the SNP-Wave

**DOI:** 10.1371/journal.pone.0102113

**Published:** 2014-07-22

**Authors:** Claudia Lamina, Margot Haun, Stefan Coassin, Anita Kloss-Brandstätter, Christian Gieger, Annette Peters, Harald Grallert, Konstantin Strauch, Thomas Meitinger, Lyudmyla Kedenko, Bernhard Paulweber, Florian Kronenberg

**Affiliations:** 1 Division of Genetic Epidemiology, Department of Medical Genetics, Molecular and Clinical Pharmacology, Innsbruck Medical University, Innsbruck, Austria; 2 Institute of Genetic Epidemiology, Helmholtz Zentrum München - German Research Center for Environmental Health (GmbH), Neuherberg, Germany; 3 Institute of Epidemiology II, Helmholtz Zentrum München – German Research Center for Environmental Health, Neuherberg, Germany; 4 Department of Molecular Epidemiology, Helmholtz Zentrum München, German Research Center for Environmental Health, Neuherberg, Germany; 5 Institute of Medical Informatics, Biometry and Epidemiology, Chair of Genetic Epidemiology, Ludwig-Maximilians-Universität, Munich, Germany; 6 Institute of Human Genetics, TechnischeUniversitätMünchen, Munich, Germany; 7 Institute of Human Genetics, Helmholtz Zentrum München – German Research Center for Environmental Health, Neuherberg, Germany; 8 First Department of Internal Medicine, Paracelsus Private Medical University Salzburg, Salzburg, Austria; Institut Jacques Monod, France

## Abstract

Structural genetic variants as short tandem repeats (STRs) are not targeted in SNP-based association studies and thus, their possible association signals are missed. We systematically searched for STRs in gene regions known to contribute to total cholesterol, HDL cholesterol, LDL cholesterol and triglyceride levels in two independent studies (KORA F4, n = 2553 and SAPHIR, n = 1648), resulting in 16 STRs that were finally evaluated. In a combined dataset of both studies, the sum of STR alleles was regressed on each phenotype, adjusted for age and sex. The association analyses were repeated for SNPs in a 200 kb region surrounding the respective STRs in the KORA F4 Study. Three STRs were significantly associated with total cholesterol (within *LDLR*, the *APOA1/C3/A4/A5/BUD13* gene region and *ABCG5/8*), five with HDL cholesterol (3 within *CETP*, one in *LPL* and one in*APOA1/C3/A4/A5/BUD13*), three with LDL cholesterol (*LDLR*, *ABCG5/8* and *CETP*) and two with triglycerides (*APOA1/C3/A4/A5/BUD13* and *LPL*). None of the investigated STRs, however, showed a significant association after adjusting for the lead or adjacent SNPs within that gene region. The evaluated STRs were found to be well tagged by the lead SNP within the respective gene regions. Therefore, the STRs reflect the association signals based on surrounding SNPs. In conclusion, none of the STRs contributed additionally to the SNP-based association signals identified in GWAS on lipid traits.

## Introduction

Plasma levels of total cholesterol, LDL cholesterol, HDL cholesterol and triglycerides are important risk factors for cardiovascular disease. They are also among the most intensely studied complex quantitative phenotypes in genetic association studies. A genome-wide meta-analysis by Teslovich et al. [1] identified 95 loci with significant influence on lipid levels. SNPs within these loci explain about 10–12% of phenotypic variance, corresponding to about 25–30% of the genetic variance. This is a rather high percentage compared to other complex quantitative phenotypes. Additional 62 variants have been identified recently [2], explaining further 1.6–2.6% depending on the phenotype. Nevertheless, a majority of the expected heritability of the lipid traits is still unexplained.

The impact of structural genetic variants has not been studied systematically, although it might contribute to some extent to the missing heritability. There are several types of structural variants that differ by the kind of variability and by their length [3]. Tandem repeats define a repetition of nucleotide motifs (2 to >1000 bp), which are concatenated adjacent to each other. Copy-number variations (CNVs) consist of repeating motifs of more than 1000 base pairs (bp). Motifs consisting of less than 10 bp are called short tandem repeats (STRs), simple sequence repeats (SSR) or microsatellites. More specifically, di-, tri-, tetra-, penta- or hexa-nucleotide repeats are the most common STRs, referring to motifs of 2, 3, 4, 5 or 6 nucleotides. A comprehensive analysis within the 1000 Genomes project estimated that tandem repeat sites occupy about 1.25% of the human genome [4]. Mutations in short tandem repeats mostly originate from insertions or deletions of repeated motifs. In such tandem repeat regions, mutation rates are very high [5]. Therefore, a high percentage of short tandem repeats are highly polymorphic and multiallelic. In consequence, STRs have been widely used in population genetic studies, genetic fingerprinting and as molecular markers for genetic association studies [6]. More specifically, STRs were used for hypothesis-free genome-wide linkage studies to derive new susceptibility loci in the era before genome-wide association studies (GWAS). They were neglected in favor of SNPs, as soon as genome-wide SNP chips became affordable and feasible. Although STRs were primarily regarded as non-functional markers in such studies, changes in length can have considerable impact on disease or disease-associated traits. For example, expansion of tandem repeats can lead to monogenetic disorders as Huntington disease or Fragile X syndrome [7]. Since STRs can also be found in promoter regions in a higher frequency than expected by chance, they might also have a high potential to modify gene regulation [8]. There is also accumulating evidence from candidate gene association studies that STRs are associated with susceptibility for complex diseases like schizophrenia, bipolar disorder, diabetes, cancer [9], asthma [10] and also cardiovascular disease [11], [12] and related intermediate phenotypes [13]. There have also been some candidate gene studies on lipid phenotypes investigating the impact of STRs. Talmud et al. [14] identified one tetranucleotide repeat within the CETP promoter that was significantly associated with LDL size, triglycerides, and apolipoprotein B concentrations. This STR was further associated with HDL cholesterol levels [15].

Common GWAS as well as the vast majority of candidate gene studies, however, do not include structural variants such as short tandem repeats. Therefore, such associations are likely missed, thereby possibly contributing to the widely discussed missing heritability [9], [16].

The intention of our study was to systematically evaluate short tandem repeats in known lipid gene regions on the lipid phenotypes total cholesterol, HDL cholesterol, LDL cholesterol and triglycerides. We hypothesized that short tandem repeats might explain an additional part of the genetic variation that is thought to be heritable. Therefore, we sorted all SNPs, which have been found in a big GWA meta-analysis to be associated with lipid phenotypes [1] by their strength of association, and selected 16 mostly unknown STRs within the genes lying near to the identified SNPs. We regressed these STRs on lipid phenotypes in two independent studies: one population-based study (KORA F4 Study) and one study in a healthy working population SAPHIR). Since a high fraction of tandem repeat polymorphisms have been shown to be well tagged by surrounding SNPs [4], [17], we also evaluated whether our selected STRs can be tagged by surrounding SNPs or if they are rather independent. Therefore, the specific questions we address are as follows: 1) Are STRs selected from known lipid candidate genes associated with the respective lipid phenotypes? 2) Are these identified STR-lipid associations independent from genome-wide significant SNPs in the respective gene regions?

## Materials and Methods

### Study populations

*The KORA F4 Study* is a population-based sample from the general population living in the region of Augsburg, Southern Germany, which has evolved from the WHO MONICA study (Monitoring of Trends and Determinants of Cardiovascular Disease). Age-and sex-stratified samples were drawn in the years 1999–2001 (n = 4,261). A total of 3,080 subjects participated in a follow-up examination in 2006/08 (KORA F4) [18], [19]. All participants are of European ancestry. Samples were available for 3063 participants.

*The SAPHIR Study* (Salzburg Atherosclerosis Prevention Program in subjects at High Individual Risk) is an observational study conducted in the years 1999–2002 involving 1770 healthy unrelated subjects (663 females and 1107 males). Study participants were recruited by health-screening programs in companies in and around the Austrian city Salzburg [20]. DNA samples and phenotypes were available for 1726 participants.

### Ethics Statement

Study participants were examined according to the principles expressed in the Declaration of Helsinki. Participants from both studies provided written informed consent. The protocol of the KORA F4 Study was approved by the Ethical Committee of the “Bayrische Landesärztekammer”, and the protocol of the SAPHIR Study was approved by the Ethical Committee of Land Salzburg. Study participant records were anonymized and de-identified prior to analysis.

### Selection of Short Tandem Repeats

First, we sorted all SNPs from the 95 loci reported in Table 1 in Teslovich et al. [1] by their p-value, starting with the lowest. For each SNP, we picked the nearest gene and retrieved the corresponding sequence +/−10 kb up- and downstream of the gene. In case of gene clusters (*APOA1/C3/A4/A5/BUD13)* or overlapping genes (*ABCG5/8*) the sequences of all genes were included. We scanned these sequences for potential short tandem repeats (STRs) using the software SERV [21] (http://www.igs.cnrs-mrs.fr/SERV). SERV identifies potential STRs in nucleotide sequences. It uses the number of repeated units, the unit length and the purity to calculate a continuous score (named “VarScore”), which provides an estimation of the repeat variability (with high scores correlating with a more pronounced variability).

**Table 1:** Gene regions for which STRs have been selected, reporting the magnitude of p-values taken from Teslovich et al. for all four investigated lipid phenotypes together with the respective lead SNP.

Gene/Gene region	STR in or near gene	Magnitude of p-value from the association with the following traits^a^				Lead-SNP^a^
		TC	LDL	HDL	TG	
*ABCG5/8*	ABCG5	10**^−45^**	10**^−47^**			rs4299376
*APOA1/C3/A4/A5/BUD13*	BUD13	10**^−57^**	10**^−26^**	10**^−47^**	10**^−240^**	rs964184
*CETP*	CETP_1	10**^−14^**	10**^−13^**	10**^−380^**	10**^−12^**	rs3764261
*CETP*	CETP_2	10**^−14^**	10**^−13^**	10**^−380^**	10**^−12^**	rs3764261
*CETP*	CETP_3	10**^−14^**	10**^−13^**	10**^−380^**	10**^−12^**	rs3764261
*FRMD5*	FRMD5				10**^−11^**	rs2929282
*HNF1A*	HNF1A	10**^−14^**	10**^−15^**			rs1169288
*HNF4A*	HNF4A	10**^−13^**		10**^−15^**		rs1800961
*JMJD1C*	JMJD1C				10**^−12^**	rs10761731
*LDLR*	LDLR	10**^−97^**	10**^−117^**			rs6511720
*LIPG*	LIPG	10**^−19^**		10**^−49^**		rs7241918
*LPA*	LPA	10**^−17^**	10**^−17^**	10**^−08^**		rs1564348 (TC, LDL), rs1084651 (HDL)
*LPL*	LPL			10**^−98^**	10**^−115^**	rs12678919
*SCARB1*	SCARB1			10**^−14^**		rs838880
*TOP1*	TOP1	10**^−17^**	10**^−19^**			rs6029526
*TRIB1*	TRIB1	10**^−36^**	10**^−29^**	10**^−19^**	10**^−55^**	rs2954029

a according to Teslovich et al. [1]; with the exception of *LPA*, the reported lead SNP in each gene region is the same for all associated lipid phenotypes.

In order to be considered for further experimental testing, a putative STR had to fulfill the following criteria: a SERV VarScore larger than 0.80, a repeat unit between 3 and 6 bp, a repeat purity larger than 97% (i.e. the percentage of sequence perfectly matching the identified repeat element) and a total repeat region length smaller than 200 bp. Dinucleotide repeats were not regarded because of their instability and the error-prone interpretation of the electrophoretic data when stutter peaks are present [22], [23]. Repeat units greater than 5 nucleotides and repeats consisting only of C's or G's were excluded because of their difficult amplification in PCR analysis.

We decided a priori to genotype 16 STRs at a maximum since this number fits two multiplex PCRs. The 54 most strongly lipid-associated genes had to be screened consecutively to obtain the first 15 STRs which matched with the above described selection criteria for STR genotyping. All gene-regions that were considered in this search for potential STRs are given in Table S1 in File S1. The STR CETP_3 was additionally selected due to its known association with lipids [14], [15], although the STR did not fulfill our bioinformatic criteria. Divergent from the prior selection criteria, we accepted a VarScore from 0.75 within *LIPG*, since the STR that actually fulfilled the criteria (VarScore = 1.76), caused unresolvable problems in the multiplex PCR and was therefore excluded. Only three out of the 15 loci were already known, two of them from forensics [24], [25]. Only one penta-nucleotide-repeat in the *LPA* gene has already been subject of genetic association analyses [12]. Table 1 lists the selected 16 STRs and the corresponding gene regions together with the identified lead SNP based on Teslovich et al. [1] and their original association results.

### Measurement of Short Tandem Repeats (STRs) - Multiplex PCR amplification

In both studies, the sixteen selected STRs were genotyped in two multiplex PCRs (see Table S2 & Figure S1 in File S1). Primers were designed with Visual OMP (DNA Software, Ann Arbor, MI) and HPLC-purified primers were purchased from Microsynth (Balgach, Switzerland). Forward primers were labeled with FAM, YY (Yakima Yellow), ATTO 550, and AT565.

The sequences were checked for existing null alleles, which could cause scoring errors and deviations from Hardy-Weinberg equilibrium by using the software Micro-Checker [26] (http://www.microchecker.hull.ac.uk/). The Hardy-Weinberg equilibrium was checked with ARLEQUIN [27] (http://cmpg.unibe.ch/software/arlequin3/).

The PCR was performed in 384 well plates in a total volume of 5 µl. The PCR mix contained 20 ng dried DNA, 2.5 µl Qiagen Multiplex PCR Plus Kit (Qiagen, Hilden, Germany), 1×Q solution (Qiagen, Hilden, Germany) and 1 µl primer-mix (final primer concentrations see Table 2). The amplification reaction for both PCRs was conducted on a DNA Engine Cycler (BioRad, Hercules, CA, USA) under following conditions: initial denaturation 95°C 15 min; 95°C 30 sec, 66°C 90 sec, 72°C 30 sec (30 cycles); final extension 68°C 30 min.

**Table 2:** Characteristics and localization of selected STRs.

STR in gene	Repeat	Position of STR according to HG build 19			Position in gene	Minimum/Median/Maximum of number of repeats in	
		Chr	Start (bp)	End (bp)		KORA F4	SAPHIR
ABCG5	AAT	2	44060592	44060473	Intron1	6/12/18	8/12/17
BUD13	TAT	11	116638744	116638685	Intron 2	6/12/18	8/12/17
CETP_1	TAT	16	56989702	56989761	∼5 kb upstream	3/9/17	3/9/16
CETP_2	TTTA	16	56997877	56997996	Intron 2	7/12/16	7/12/16
CETP_3	GAAA	16	56993722	56993961	promoter	37/48/56	38/48/56
FRMD5	GAT	15	44379990	44379931	Intron 1	11/15/20	11/15/20
HNF1A	ATCT	12	121425538	121425657	Intron 1	7/11/14	8/11/14
HNF4A	TTAT	20	42982780	42982839	∼1.5 kb upstream	6/10/13	7/10/12
JMJD1C	TTG	10	65151334	65151215	Intron 1	8/12/15	8/12/14
LDLR	TTG	19	11202856	11202915	Intron 1	7/10/18	7/10/17
LIPG	AATA	18	47083781	47083900	∼5 kb upstream	5/9/11	5/9/11
LPA	TTTTA	6	161086738	161086619	Intron 1	5/8/12	4/8/11
LPL	TTAT	8	19815455	19815574	Intron 6	6/11/14	7/11/14
SCARB1	AAAGA	12	125255821	125255702	∼6 kb downstream	5/10/28	5/10/28
TOP1	AAAT	20	39688036	39688095	Intron 2	7/12/17	8/12/16
TRIB1	AAAC	8	126440163	126440222	∼2 kb upstream	7/11/14	7/11/14

### Measurement of Short Tandem Repeats (STRs) - Electrophoresis and data analysis

For the electrophoresis 1 µl PCR product was diluted 1∶20, mixed with 8.8 µl HiDiformamide and 0.2 µl GeneScan-500 LIZ size standard (all Applied Biosystems, Foster City, CA) and denatured at 95°C for 5 minutes. The electrophoresis was run on an ABI 3730 s Genetic Analyzer using POP-7 polymer (both Applied Biosystems). Data were analyzed using GeneMapper-Software, version 4.1 (Applied Biosystems) (Figure S1 a and b in File S1). For each STR at least two samples were subjected to Sanger sequencing to calibrate the allele calling algorithm in GeneMapper-Software.

### SNP genotyping and imputation in KORA F4

For 2940 participants of the KORA F4 Study, a genome-wide SNP chip is available (Affymetrix Axiom). Quality control criteria for genotypes entering the genotype imputation were at least 97% call rate per person and 98% callrate per SNP, HWE (p-value ≥5×10−6) and a minor allele frequency of ≥0.01. Genotypes were imputed with the software IMPUTE (IMPUTE v2.3.0) based on the 1000 g phase 1 reference panel (all populations, 1000 G integrated phase 1 vers 3, March 2012) [28]. Therefore, a high density of SNPs was available for analysis in KORA F4 for each of the selected gene-regions. The specific gene regions were defined by the lead SNP according to Teslovich et al. (Table 1) ±100 kB. All SNPs lying within these regions with a MAF of >1% and imputation quality (info)>0.6 were extracted from the imputed genome-wide dataset. Genomic positions and LD refer to HG build 19 (1000 Genomes, phase 1 vers 3, March 2012).

### Measurement of lipids

In KORA F4, total cholesterol (TC) was determined by cholesterol-esterase method (CHOL Flex, Dade-Behring, Germany), triglycerides (TG) and HDLC using the TGL Flex and AHDL Flex method (Dade-Behring), respectively, and LDLC was measured by a direct method (ALDL, Dade-Behring) [29].

All participants were fasting for at least 8 hours. For the present analysis, all participants taking lipid-lowering drugs were excluded. The analysis dataset in KORA F4 is thus based on 2553 participants with available STR measurements and genotypes derived from genome-wide SNP-chips and imputation.

In SAPHIR, blood samples were collected after an overnight fasting period. A complete lipoprotein profile including fasting TC, TG, HDLC and LDLC was determined using routine laboratory procedures (Roche Diagnostics GmbH, Mannheim, Germany). For statistical analysis, all participants taking lipid-lowering drugs were excluded. The analysis dataset in the SAPHIR Study is therefore based on 1648 participants.

### Statistical methods

For each STR and individual, full allelic data was available. The association analysis was based on both, the allele-specific information as well as the sum of both alleles as the explaining variable. To account for the fact that there are two independent alleles from each individual in the allele-specific analysis, a robust standard error using a sandwich variance-covariance-matrix (function sandcov, R-package haplo.ccs) was calculated to derive the p-values for these analyses. Table 1 shows all loci together with the respective STRs that have been measured within these loci. Most of these loci have been shown to be associated with more than one lipid phenotype in Teslovich et al. and are evaluated on these same phenotypes in the present investigation: from the 16 measured STRs, 12 were regressed on total cholesterol, 10 on HDL cholesterol, 10 on LDL cholesterol and 8 on triglycerides. In KORA F4 and the SAPHIR Study, linear regression analyses were performed, regressing the sum of STR alleles on the respective lipid phenotypes. A linear mixed model assuming random intercepts was used to combine both datasets and derive a common effect estimate for each STR. Using Bonferroni correction, a p-value smaller than 0.05/40 = 0.00125 was defined to be significant, accounting for the number of STRs studied in all phenotypes (40 = 12+10+10+8).

To compare the STR results with the SNPs within each specific gene region, all SNPs in a region ±100 kB around the lead SNP were extracted from the imputed genotype data in KORA F4 and regressed on the respective lipid phenotype of interest. An additive inheritance model was assumed for each SNP. Triglyceride levels were ln-transformed for all analyses due to the highly skewed distribution. All linear and linear mixed models were adjusted for age and sex.

To evaluate whether STRs are associated with the lipid phenotypes independently from the respective lead SNPs, the regression models from STRs were additionally adjusted for this same lead SNP. Pearson correlation coefficient r^2^ is given as a measure of LD between STRs and SNPs.

Association analyses of SNPs were performed in SNPTest v.2.5 [30], all other analyses in R 3.0.1. The program LocusZoom [31] was used to create regional association plots for gene regions of interest.

## Results

### STR characteristics

The distribution of the STRs varies from 3 to 20 repeats, with the exception of CETP_3 (37–56 repeats). Figure S2 in File S1 shows a comparable distribution of the number of repeats based on alleles as well as on the sum of alleles for all STRs in the SAPHIR and KORA F4 studies. Minimum, median and maximum numbers of repeats for both studies are provided in Table 2.

### STR association results with lipid phenotypes

All STR association results for KORA F4, SAPHIR and both studies combined are given in the Tables S3a)–d) in File S1. Since there was hardly any difference between allele-specific analysis and analysis based on sum of alleles, the specific allelic information was discarded in favor of a denser representation taking the sum of both alleles in all subsequent analyses. For all four studied phenotypes, significant associations with STRs were observed for all four studied phenotypes (Table 3). In the combined analysis of KORA F4 and SAPHIR, three STRs were significantly associated with total cholesterol (LDLR, p = 6.23E-07; BUD13, p = 1.42E-05; ABCG5, p = 6.51E-05), five with HDL cholesterol (CETP_1, p = 7.62E-12; CETP_2, p = 2.19E-14; CETP_3, p = 1.35E-28; LPL, p = 1.08e-05, BUD13, p = 1.43E-04), three with LDL cholesterol (LDLR, p = 9.96E-09; ABCG5, p = 4.70E-05; CETP_1, p = 2.32E-05) and two with triglycerides (BUD13, p = 1.04E-15; LPL, p = 5.34E-04).

**Table 3:** Results of linear mixed models and linear models on the investigated lipid phenotypes for all STRs which are significantly associated with lipids in KORA F4 and SAPHIR combined: 1) regression of the sum of STR alleles on lipids in KORA F4 and SAPHIR combined, 2) regression of the sum of STR alleles on lipids in SAPHIR, 3) regression of the sum of STR alleles on lipids in KORA F4, 4) regression of the minor allele using the lead SNP (LS) in the gene region on lipids in KORA F4, 5) regression of the minor allele using the best SNP in the gene region (LS+/−100 kB) on lipids in KORA F4.

STR/gene	SAPHIR & KORA F4		SAPHIR study		KORA F4 study		KORA F4 study		r^2^ between lead SNP^a^ and STR	KORA F4 study	
	STR (sum of alleles)		STR (sum of alleles)		STR (sum of alleles)		Lead SNP^a^			STR, adjusted for lead SNP	
	beta	p-value	beta	p-value	beta	p-value	beta	p-value		beta	p-value
**Total Cholesterol**											
LDLR	−1.3585	6.23E-07	−1.1168	0.0232	−1.4730	6.27E-06	−8.0470	2.01E-06	0.8660	−0.4522	0.6111
BUD13	1.2125	1.42E-05	1.8036	1.04e-04	0.8646	0.0132	5.4035	3.56E-04	0.5668	−0.0798	0.8800
ABCG5	1.2864	6.51E-05	1.9165	1.93e-04	0.8436	0.0417	2.5913	0.0234	0.1556	0.5433	0.2277
**LDL Cholesterol**											
LDLR	−1.4080	9.96E-09	−1.0408	0.0218	−1.5796	4.79E-08	−8.689	7.01E-09	0.8660	−0.4325	0.5831
ABCG5	1.1806	4.70E-05	1.6747	4.05e-04	0.8251	0.0246	1.8916	0.0622	0.1556	0.6331	0.1128
CETP_1	0.6998	2.32E-05	0.6395	0.0199	0.7400	3.36E-04	−2.2240	0.0265	0.3008	0.7265	0.0033
**HDL Cholesterol**											
CETP_1	−0.4513	7.62E-12	−0.3773	5.59e-04	−0.5014	1.15E-09	4.0230	3.67E-24	0.3008	−0.0715	0.4610
CETP_2	0.5595	2.19E-14	0.5267	1.14e-05	0.5798	3.37E-10	4.0230	3.67E-24	0.0463	0.4015	1.55e-05
CETP_3	−0.3393	1.35E-28	−0.3451	4.36e-11	−0.3359	3.33E-19	4.0230	3.67E-24	0.8259	−0.0085	0.9240
LPL	0.6805	1.08E-05	0.7685	0.0027	0.6140	0.0015	1.8011	0.0042	0.1609	0.4706	0.0259
BUD13	−0.3824	1.43E-04	−0.7041	3.60e-05	−0.1887	0.1278	−1.2328	0.0216	0.5668	0.0275	0.884
**ln(Triglycerides)**											
BUD13	0.0311	1.04E-15	0.0408	2.46e-10	0.0252	2.02E-07	0.1532	3.16e-13	0.5668	−0.0011	0.8810
LPL	−0.0208	5.34E-04	−0.0272	0.0052	−0.0167	0.0285	−0.0963	9.88e-05	0.1609	−0.0059	0.4772

All analyses are adjusted for age and sex.

a according to Teslovich et al. [1]; beta effect estimate for the lead SNP refers to the minor allele, assuming an additive model.

### Comparing association results based on STRs with those based on SNPs

To put these findings into context, they were compared with results of SNP-association analyses in the respective gene regions. These analyses are only available in KORA F4, since no SNP microarray has been genotyped in SAPHIR. Table 3 shows the significantly associated STRs compared to the results for the respective lead SNP according to Teslovich et al [1]. For most loci given in Table 3, the lead SNPs were at least nominally significantly associated with their respective lipid phenotypes. Detailed results for all significantly and non-significantly associated STRs and the lead SNPs in the gene regions are given in Tables S4a)–d) in File S1. In general, none of the investigated STRs yielded a substantially lower p-value and therefore higher association peak than the lead SNP within their gene regions. For all significantly associated STRs, regional plots were created to further evaluate the association and LD structure of the SNPs within the gene regions of interest. The results of the STRs on KORA F4 were added to these plots to put them in context (Figures S3a)–k) in File S1). Association results of STRs adjusted for the lead SNP are given in Table 3 as well as Tables S4a)–d) in File S1.

**Figure 1:**
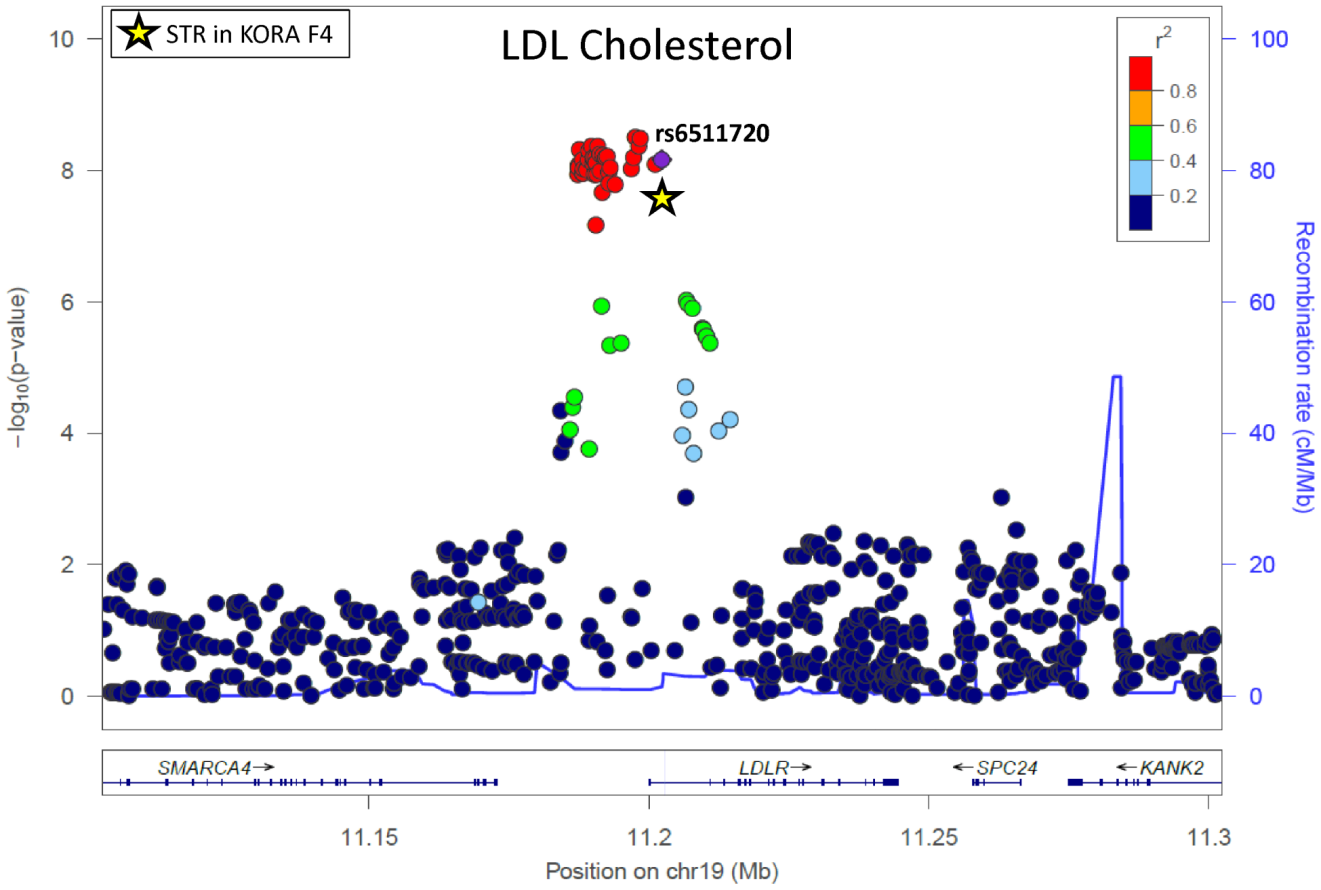
Regional plot showing the association of SNPs/STR in the *LDLR* region with LDL cholesterol. LD refers to the lead SNP according to Teslovich et al. [1] (rs6511720); p-values of the STR in KORA F4 is marked as a star.

**Figure 2:**
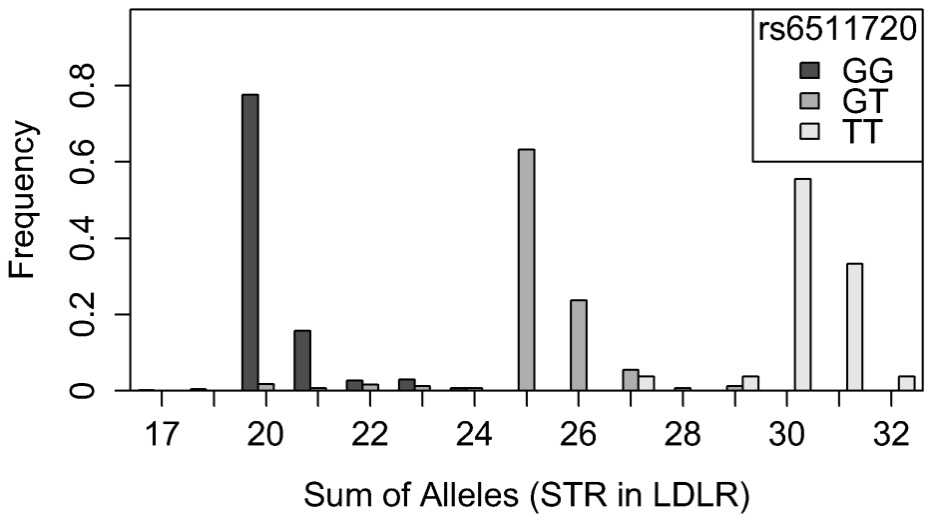
Distribution of sum of *LDLR*-STR-alleles, separated for rs6511720 genotypes.

For *LDLR*, for example, the STR falls in exactly the same genetic region as the conglomeration of SNPs constituting the highest association peak for total and LDL cholesterol (Figure 1). The assumption that the lead SNP rs6511720 tags the STR in *LDLR* can be verified by Figure 2: individuals with the common genotype GG generally have low repeat numbers. With increasing repeat numbers the number of rare alleles of that SNP increases. The correlation coefficient r^2^ between this STR and rs6511720 is 0.866. Although the STR was highly associated with total cholesterol (p = 6.27E-06 in KORA F4) and LDL cholesterol (p = 4.79E-08 in KORA F4) there was no significant association anymore after adjusting for the lead SNP (p = 0.611, p = 0.5831).

**Figure 3:**
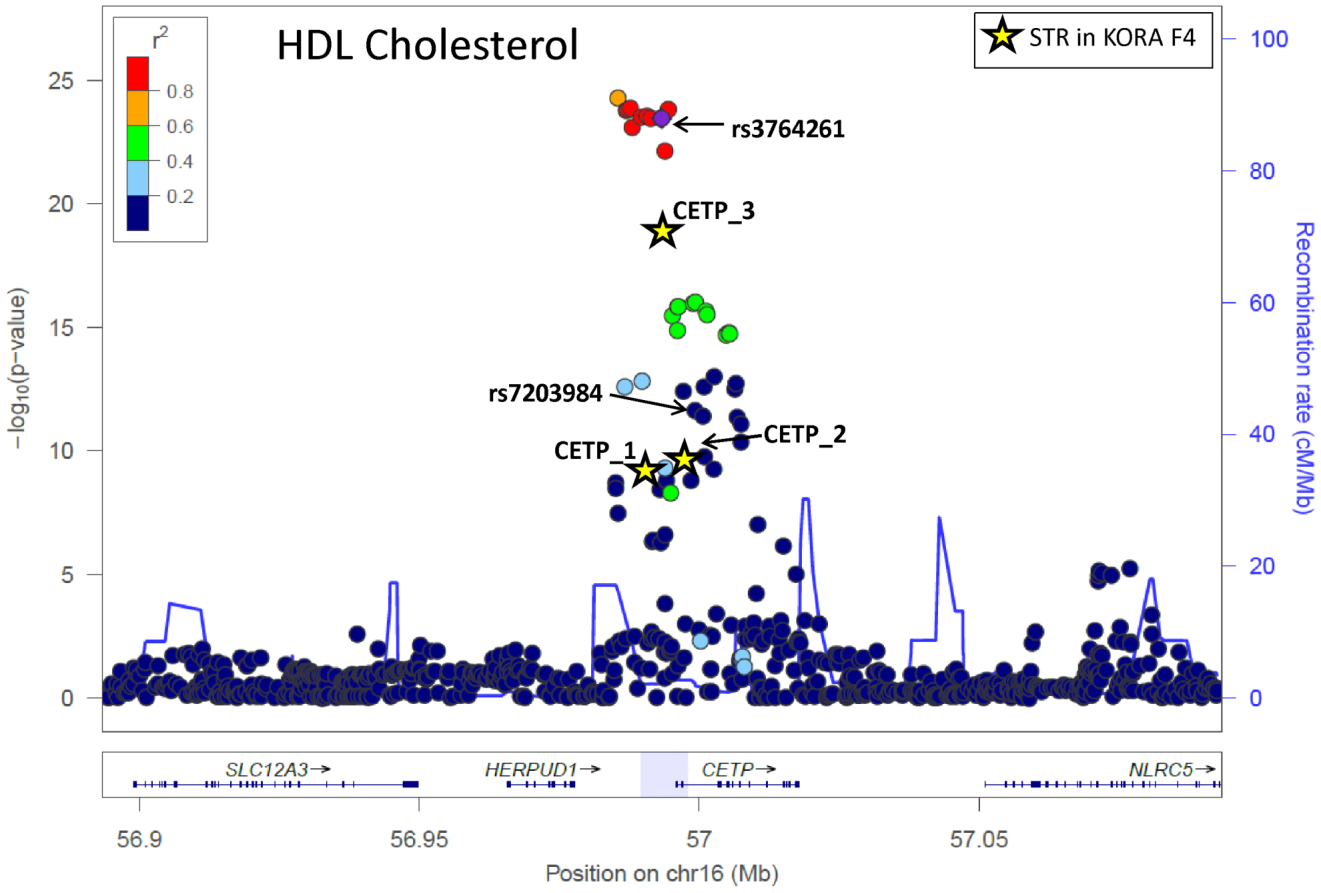
Regional plot showing the association of SNPs/STR in the *CETP* region with HDL cholesterol. LD refers to the lead SNP according to Teslovich et al. [1] (rs3764261); p-values of STRs in KORA F4 are marked as stars.

**Figure 4:**
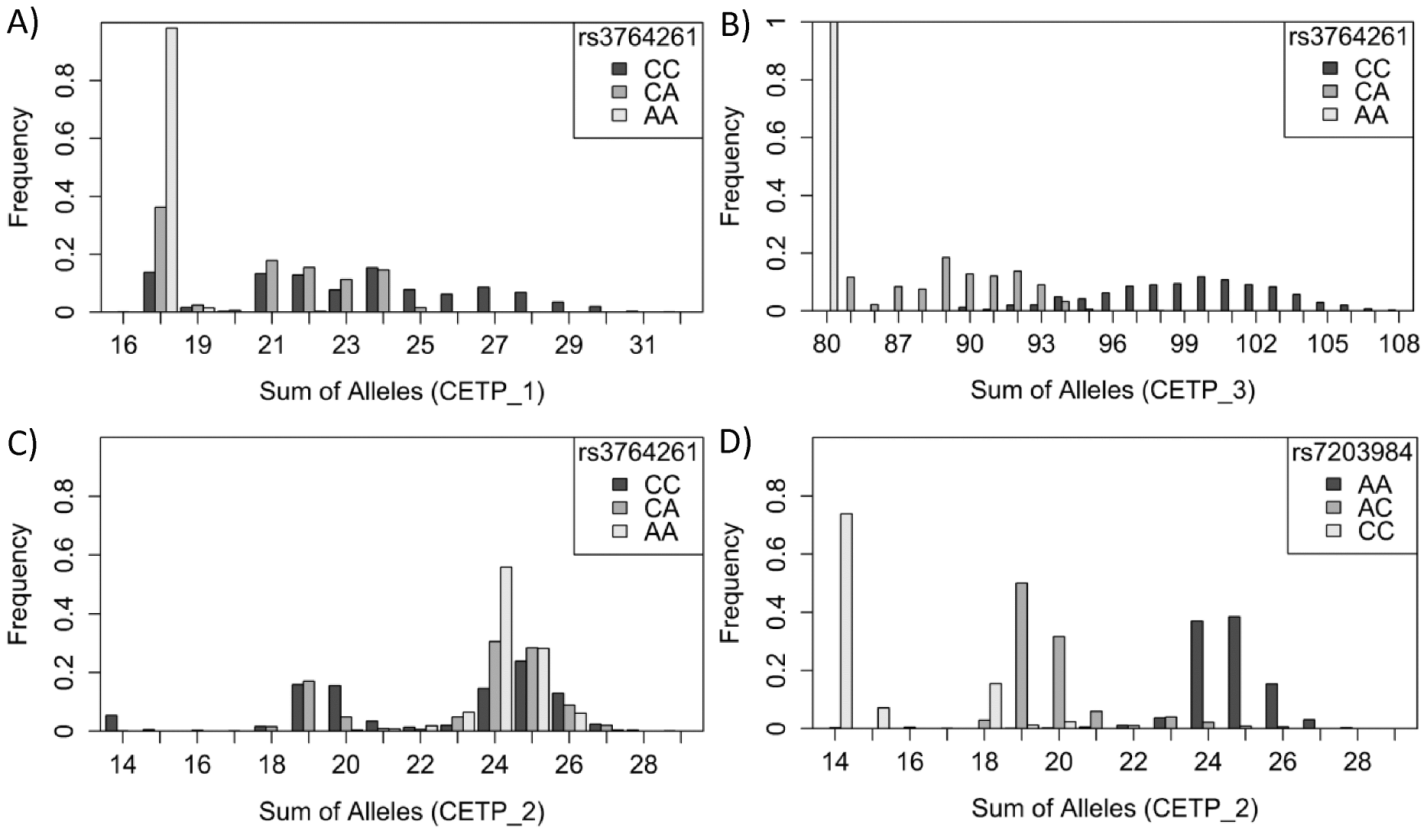
Distribution of sum of STR-alleles, separated for genotypes: A) Distribution of CETP_1, separated for rs3764261 genotypes, B) Distribution of CETP_3, separated for rs3764261 genotypes, C) Distribution of CETP_2, separated for rs3764261 genotypes, D) Distribution of CETP_2, separated for rs7203984 genotypes.

For the *CETP* gene region, three STRs have been selected. All three of them are significantly associated with HDL cholesterol. However, the STRs pick up the association signal of their surrounding SNPs (Figure 3). CETP_1 is only marginally correlated with the lead SNP rs3764261 (r^2^ = 0.3008, Figure 4A)). Still, the association signal with HDL cholesterol (p = 1.15E-09) vanishes after adjusting for that SNP (p = 0.4610). This same pattern can also be observed for CETP_3 which is highly correlated with the lead SNP (r^2^ = 0.8259, Figure 4B)). All individuals with AA genotype are homozygote with repeat length 40 (sum of repeat length 80), for CA genotype sum of repeat length varies between 85 and 95, for CC genotype between 90 and 108. Thus, after adjustment for that SNP, the p-value of association vanishes from p = 3.33E-19 to p = 0.9240. CETP_2, however, seems to be independent from rs3764261 (r^2^ = 0.0463). This STR is still significantly associated with HDL cholesterol after adjusting for the lead SNP (p = 1.55E-05). From the regional plot (Figure 3) one might speculate that CETP_2 is tagged by the cluster of SNPs, which are independent from the lead SNP and are directly located downstream of CETP_2. To confirm this assumption, we set up a list of independent SNPs from the lead SNP (r^2^<0.1) and selected the SNP with the lowest p-value with HDL cholesterol. This SNP (rs7203984, p = 2.48E-12) represents this cluster of SNPs directly next to CETP_2 and is highly correlated with this STR (r^2^ = 0.8471, see Figure 4D). After adjusting for rs7203984, the significant association of CETP_2 with HDL cholesterol (3.37E-10) is completely absent (β = −0.0672, p = 0.7747).

For all other STRs, which were shown to be significantly associated with the lipid phenotypes, similar observations can be made: The distribution of STRs depends on the genotypes of the respective lead SNPs (Figures S4 a)–h) in File S1). Therefore, after adjusting for the lead SNP, effect estimates are diminished and there are no significant associations of STRs with lipid phenotypes left (Table 3).

The published lead SNPs taken from Teslovich et al. [1] are not necessarily the SNPs with the lowest p-value in the KORA F4 study. All analyses were repeated based on the best SNP in KORA F4 within each extracted gene region. The best SNPs in KORA F4 are in high LD with the lead SNPs in the relevant gene regions (Figure S2 in File S1), and therefore, as expected, results did not change.

## Discussion

Based on a published genome-wide search for lipid loci, we systematically selected STRs in or near genes at the most significant association peaks. We compared the association signals in these STRs with the association signals from SNPs genotyped by GWAS microarrays from the same gene regions. None of the investigated STRs, however, was significantly associated after adjusting for the lead SNPs within that gene region. Since these STRs have been found to be well tagged by SNPs, the STRs reflect the association signals based on surrounding SNPs. Therefore, none of the STRs contributed additionally to the SNP-based association signals.

In our analysis, three STRs were significantly associated with total cholesterol, five with HDL cholesterol, three with LDL cholesterol and two with triglycerides. We could replicate the association of the known STR in the CETP promoter region with HDL [15], [32], but it was only nominally associated with LDL cholesterol and triglycerides. The other significant associations are all novel and have not been described elsewhere. The only other known STR from the literature within *LPA* did not yield any significant results with lipids in our investigation. This STR has been shown to be highly associated with lipoprotein(a) in the past [12], [33], but not with any other phenotypes.

The finding of significant associations between STRs and lipids imposes the question, whether SNP-based analyses would have led to the same results. The significantly associated STRs are located within a high-LD-block with about the same p-value as these surrounding SNPs. This is especially the case for the STRs within *LDLR* (on total cholesterol and LDL cholesterol) and *CETP* (on HDL cholesterol). Additional adjustment for the lead SNP resulted in attenuation of STR association with the lipid phenotypes. Consequently, except CETP_2, none of the STRs was significantly associated anymore. Although CETP_2 was not correlated with the respective lead SNP in that region, it was correlated with an independent SNP in that gene region, representing the second highest association signal in *CETP*. Since this SNP and the adjacent SNP cluster was still genome-wide significant, these STR-tagging SNPs would have been identified in a genome-wide analysis based on SNPs anyway. Therefore, none of the STRs contributed additionally to the SNP-based association signals.

We cannot, however, clarify whether the STRs trigger the association of SNPs in the respective gene regions or the other way round. In a comprehensive and systematic investigation of 179 human genomes within the 1000 Genomes project, it was shown that 41% of tandem repeat polymorphisms are tagged by at least one SNP (with r^2^>0.8) [4]. Therefore, it might be expected that a fair proportion of STRs drive the results of GWAS on common phenotypes.

The strength of our investigation is the systematic selection process of STRs in known lipid candidate genes. This approach took the location of the STRs in and around the respective candidate gene into account, their variability and a high repeat purity. To our knowledge, such an approach has not been reported before. So far, there was more interest in finding SNPs that are able to tag known associated STRs, since genotyping technologies for SNPs are easier and cheaper on a large scale.

It was surprising that STRs fulfilling our selection criteria could only be found in about 26% of the examined genes. Therefore, this approach cannot be imposed on any specific gene of interest. However, our selection strategy seems to follow to great extent what would have been expected from a random sample of polymorphic STRs. According to Payseur et al. [34], who examined two complete genome sequences, tetranucleotide repeats are the most variable tandem repeat polymorphisms, followed by di- and pentanucleotide repeats. Trinucleotide polymorphisms were shown to be the least variable polymorphisms. With our search strategy we selected tetra-nucleotide repeats most frequently (8×), followed by trinucleotide repeats (6×) and pentanucleotide repeats (2×). We excluded dinucleotide repeats due to the error-prone typing of this repeats class. Comparable to Payseur et al. [34], most repeat numbers varied between 3 and 20 with only one exception, that is the knowledge-driven selection of the third STR in the *CETP* gene region. Trinucleotide repeats seem to be overrepresented, though, which is a consequence of the selection strategy: a high VarScore and high purity both favor a lower number of bases in the repeated regions.

Another strength of our investigation is that two independent studies, one population-based, one based on a healthy working population, were used to decrease false-positive findings. In one of these studies (KORA F4), 1000-Genomes-based imputed genotypes were available to compare and adjust the association results of STRs with surrounding SNPs. Unfortunately, we did not have SNP genotype data for the SAPHIR study available.

The necessity to restrict our evaluation to 16 STRs represents a major limitation of this work. A comprehensive analysis of all genome-wide significant regions originally identified in [1] was not possible due to the current technological limitations. Therefore, our results cannot be transferred directly to other STRs, other gene regions or other phenotypes. Nevertheless, in our study we covered already more than half of the most strongly lipid-associated genes. For many genes, however, no putative STRs according to our selection criteria were detected within the gene +/−10 kB. This 10 kb cut-off, although necessarily arbitrary, was based on the assumption that this shall capture most promoter elements. For example, the ENCODE project [35] reported symmetrical presence of transcription factors +/−5 kb around the transcription factor binding site. In general, the potential impact of STRs on gene regulation is well-established. A well-known example illustrating the progressive disruption of regulatory elements by STR copies, is the so-called UGT1A1*28 polymorphism. This polymorphism consists of a TA repeat located exactly in the TATAA- element of the UGT1A1 promoter and increases the bilirubin levels in blood by reducing the UGT1A1 expression [13], [36], [37]. Our idea was to capture such STRs which have potential functional consequences affecting cis regulatory regions in proximity of the gene. However, our approach does not provide information for STRs affecting intergenic regulatory elements.

To conclude, from the 16 systematically selected STRs within known lipid susceptibility genes, several were highly associated with their respective phenotypes. However, all significantly associated STRs were well tagged by their surrounding SNPs. Thus, none of these STRs contributed additionally to the SNP-based association signal. Although STRs and other structural variants are neglected in SNP-based association studies and therefore, their associations are likely missed, this is not the case in our investigation based on known susceptibility genes on total cholesterol, HDL cholesterol, LDL cholesterol and triglycerides.

## Supporting Information

File S1 (PDF)
