# Supplementary material for: A Systematic Evaluation of Short Tandem Repeats in Lipid Candidate Genes: Riding on the SNP-Wave
Source: PLoS One. 2014 Jul 22;9(7):e102113. doi: 10.1371/journal.pone.0102113 (PMC4106801; doi:10.1371/journal.pone.0102113)

Supporting Information to

A systematic evaluation of short tandem repeats in lipid candidate genes: riding on the SNP-wave

Claudia Lamina[1], Margot Haun[1], Anita Kloss-Brandstätter[1], Stefan Coassin[1], Christian Gieger[2], Annette Peters[3], Konstantin Strauch[2,4], Thomas Meitinger[5,6], Lyudmyla Kedenko[7], Bernhard Paulweber[7], Florian Kronenberg[1]

[1] Division of Genetic Epidemiology, Department of Medical Genetics, Molecular and Clinical Pharmacology, Innsbruck Medical University, Innsbruck, Austria

[2] Institute of Genetic Epidemiology, Helmholtz Zentrum München - German Research Center for Environmental Health (GmbH), Neuherberg, Germany

[3] Institute of Epidemiology II, Helmholtz Zentrum München – German Research Center for Environmental Health, Neuherberg, Germany

[4] Institute of Medical Informatics, Biometry and Epidemiology, Chair of Genetic Epidemiology, Ludwig-Maximilians-Universität, Munich, Germany

[5] Institute of Human Genetics, Technische Universität München, Munich, Germany

[6] Institute of Human Genetics, Helmholtz Zentrum München – German Research Center for Environmental Health, Neuherberg, Germany

[7] First Department of Internal Medicine, Paracelsus Private Medical University Salzburg, Austria

Corresponding Author:
Florian Kronenberg, MD
Division of Genetic Epidemiology
Department of Medical Genetics, Molecular and Clinical Pharmacology,
Innsbruck Medical University
Schöpfstr. 41, A-6020 Innsbruck, AUSTRIA
Phone: (+43)512-9003-70560, Fax: (+43)512-9003-73560
E-mail: Florian.Kronenberg@i-med.ac.at

Table of contents

Table S1: All Gene-regions (gene +/- 10 kb flanking region) that were searched for potential STRs sorted by their p-value in Teslovich et al. (Teslovich et al., 2010); the length of the gene region is given together with the number of potential STRs (DNA regions showing STR-like sequence structures) and the number of selected putative STRs, which were selected based on the described selection criteria.

Figure S1: Multiplex PCR amplification of the STRs. Electropherogram of both multiplex PCRs (Figure S1a: PCR1, Figure S1b: PCR2), each containing 8 STR loci after the electrophoresis on the ABI 3700s Genetic Analyzer.

Figure S2: Distribution of number alleles and sum of alleles for each STR. In A) SAPHIR and B) KORA F4

Table S2: Primer sequences, concentration and labelling for PCR amplification

Table S3:
Association of sum of alleles and single alleles in each STR with Cholesterol phenotypes in SAPHIR, KORA and both studies combined, all age and sex-adjusted, excluding those individuals with lipid-lowering drugs; STRs are sorted by their p-value in Teslovich et al. (Teslovich et al., 2010)

- **a)** Results for Total cholesterol
- **b)** Results for HDL cholesterol
- **c)** Results for LDL cholesterol
- **d)** Results for ln-transformed Triglycerides

Table S4:
Regression on Cholesterol phenotypes in KORA F4: Comparing the results for the sum of alleles of STRs, for the lead SNP within that gene region (= best SNP in Teslovich et al. (Teslovich et al., 2010)) and for the sum of alleles of STRs, adjusted for the lead SNP (this latter analysis only given for STRs, which were significantly associated with lipids in the combined analysis in SPHIR & KORA F4).. All regression models were adjusted for age and sex, excluding those individuals with lipid-lowering drugs; STRs are sorted by their p-value in Teslovich et al. (Teslovich et al., 2010)

- **a)** Results for Total cholesterol
- **b)** Results for HDL cholesterol
- **c)** Results for LDL cholesterol
- **d)** Results for ln-transformed Triglycerides

Figure S3:

Regional plots showing the association of SNPs and STRs in

a) the *LDLR* gene region on Total Cholesterol
b) the *APOA1/C3/A4/A5/BUD13* gene region on Total Cholesterol
c) the *ABCG5/8* gene region on Total Cholesterol
d) the *CETP* gene region on HDL Cholesterol
e) the *LPL* gene region on HDL Cholesterol
f) the *APOA1/C3/A4/A5/BUD13* gene region on HDL Cholesterol
g) the *LDLR* gene region on LDL Cholesterol
h) the *ABCG5/8* gene region on LDL Cholesterol
i) the *CETP* gene region on LDL Cholesterol
j) the *APOA1/C3/A4/A5/BUD13* gene region on Triglycerides
k) the *LPL* gene region on Triglycerides

LD refers to the lead SNP according to Teslovich et al. (Teslovich et al., 2010); p-value of STR in KORA F4 are marked as stars.

Figure S4:

Distribution of sum of STR-alleles, in groups of genotypes of the lead SNP according to Teslovich et al. (Teslovich et al., 2010). Frequencies sum up to one in each genotype group. Black bars: STR frequencies of individuals with homozygote common genotype; grey bars: heterozygotes; light grey bars: homozygote rare genotype

a) Distribution of STR in LDLR, separated for rs6511728 genotypes
b) Distribution of STR in BUD13, separated for rs964184 genotypes
c) Distribution of STR in ABCG5, separated for rs4299376 genotypes
d) Distribution of STR_1 in CETP, separated for rs3764261 genotypes
e) Distribution of STR_2 in CETP, separated for rs3764261 genotypes
f) Distribution of STR_2 in CETP, separated for rs7203984 genotypes
g) Distribution of STR_3 in CETP, separated for rs3764261 genotypes
h) Distribution of STR in LPL, separated for rs12678919 genotypes

Table S1: All gene-regions (gene +/- 10 kb flanking region) that were searched for potential STRs sorted by their p-value in Teslovich et al. (Teslovich et al., 2010); the length of the gene region is given together with the number of potential STRs (DNA regions showing STR-like sequence structures) and the number of selected putative STRs, which were chosen based on the described selection criteria.

Gene	p-value in Teslovich	Gene (+/- 10 kb flanking)	Length	# potential STRs	# selected putative STRs
CETP	0.0E+00	chr 16: 56985762 - 57027757	41,995	55	2
BUD13, ZNF259, APOA5	7.0E-240	chr 11: 116608886 - 116673136	64,250	46	1
APOA4	7.0E-240	chr 11: 116681419 - 116704022	22,603	23	0
APOA1	7.0E-240	chr 11: 116696469 - 116718338	21,869	18	0
SORT1	1.0E-170	chr 1: 109842192 - 109950573	108,381	69	0
APOE, APOC1	9.0E-147	chr 19: 45399011 - 45432606	33,595	33	0
GCKR	6.0E-133	chr 2: 27709709 - 27756551	46,842	43	0
LDLR	4.0E-117	chr 19: 11191275 - 11254492	63,217	60	1
LPL	2.0E-115	chr 8: 19786482 - 19834769	48,287	23	1
APOB	4.0E-114	chr 2: 21214301 - 21276945	62,644	26	0
LIPC	3.0E-96	chr 15: 58692768 - 58871072	178,304	92	0
MLXIPL	6.0E-58	chr 7: 72997524 - 73048873	51,349	52	0
TBL2	6.0E-58	chr 7: 72973262 - 73003121	29,859	20	0
BCL7B	6.0E-58	chr 7: 72940686 - 72982332	41,646	25	0
TRIB1	3.0E-55	chr 8: 126432563 - 126460647	28,084	25	1
LIPG	3.0E-49	chr 18: 47078401 - 47129272	50,871	45	1
ABCG5, ABCG8	2.0E-47	chr 2: 44029611 - 44115605	85,994	45	1
HMGCR	9.0E-47	chr 5: 74622154 - 74667929	45,775	27	0
ANGPTL3	9.0E-43	chr 1: 63053158 - 63081830	28,672	16	0
CILP2	3.0E-38	chr 19: 19639074 - 19667468	28,394	11	0
ABCA1	2.0E-33	chr 9: 107533283 - 107700518	167,235	95	0
LCAT	8.0E-33	chr 16: 67963653 - 67988034	24,381	14	0
PCSK9	2.0E-28	chr 1: 55495221 - 55540525	45,304	26	0
TIMD4	7.0E-28	chr 5: 156336293 - 156400266	63,973	49	0
PPP1R3B	6.0E-25	chr 8: 8983765 - 9018206	34,441	22	0
HPR	3.0E-24	chr 16: 72078522 - 72121145	42,623	18	0
FADS1, FADS2, FADS3	5.0E-24	chr 11: 61557099 - 61669006	111,907	58	0
PLTP	2.0E-22	chr 20: 44517399 - 44550794	33,395	22	0
GALNT2	4.0E-21	chr 1: 230192956 - 230427870	234,914	108	0
TOP1	4.0E-19	chr 20: 39647458 - 39763127	115,669	53	1
HLA-DRA	4.0E-19	chr 6: 32397619 - 32422823	25,204	13	0
LRP4	3.0E-18	chr 11: 46868419 - 46950173	81,754	67	0
LPA	2.0E-17	chr 6: 160942515 - 161097407	154,892	56	1
LILRA3	4.0E-16	chr 19: 54789854 - 54819952	30,098	15	0
HNF4A	1.0E-15	chr 20: 42974340 - 43071485	97,145	78	1
KLF14	1.0E-15	chr 7: 130407401 - 130428888	21,487	23	0
ST3GAL4	1.0E-15	chr 11: 126263311 - 126294533	31,222	13	0
HLA-C	2.0E-15	chr 6: 31226526 - 31249863	23,337	9	0

MVK	7.0E-15	chr 12: 110001060 - 110045067	44,007	28	0
HNF1A	1.0E-14	chr 12: 121406346 - 121450315	43,969	20	1
SCARB1	3.0E-14	chr 12: 125251402 - 125377214	125,812	17	1
IRF2BP2	5.0E-14	chr 1: 234730015 - 234755271	25,256	16	0
NAT2	5.0E-14	chr 8: 18238755 - 18268728	29,973	11	0
STARD3	1.0E-13	chr 17: 37783318 - 37829737	46,419	8	0
PLEC1	4.0E-13	chr 8: 144979321 - 145060902	81,581	36	0
ABO	6.0E-13	chr 9: 136115788 - 136160617	44,829	22	0
MOSC1	6.0E-13	chr 1: 220950101 - 220997730	47,629	26	0
CYP7A1	2.0E-12	chr 8: 59392737 - 59422795	30,058	18	0
TTC39B	3.0E-12	chr 9: 15153620 - 15317358	163,738	27	0
JMJD1C	3.0E-12	chr 10: 64916981 - 65235722	318,741	116	1
*BRAP**	7.0E-12	chr 12: 112071886 - 112133524	61,638	28	0*
*KLHL8**	9.0E-12	chr 4: 88073674 - 88151420	77,746	59	0*
MYLIP	1.0E-11	chr 6: 16119356 - 16157112	37,756	10	0
*FRMD5**	2.0E-11	chr 15: 44152962 - 44497429	344,467	66	1

**STRs in BRAP and KLHL8 fulfilled the selection criteria, but have been discarded in favor of FRMD5, since Varscore in FRMD5 indicated a higher variability (1.1) than in BRAP or KLHL8 (0.94) with almost equal p-value in the original paper Teslovich et al., 2010.*

Figure S1a: Multiplex PCR amplification of the STRs: Electropherogram of the multiplex PCR 1 containing 8 STR loci after the electrophoresis on the ABI 3700s Genetic Analyzer.

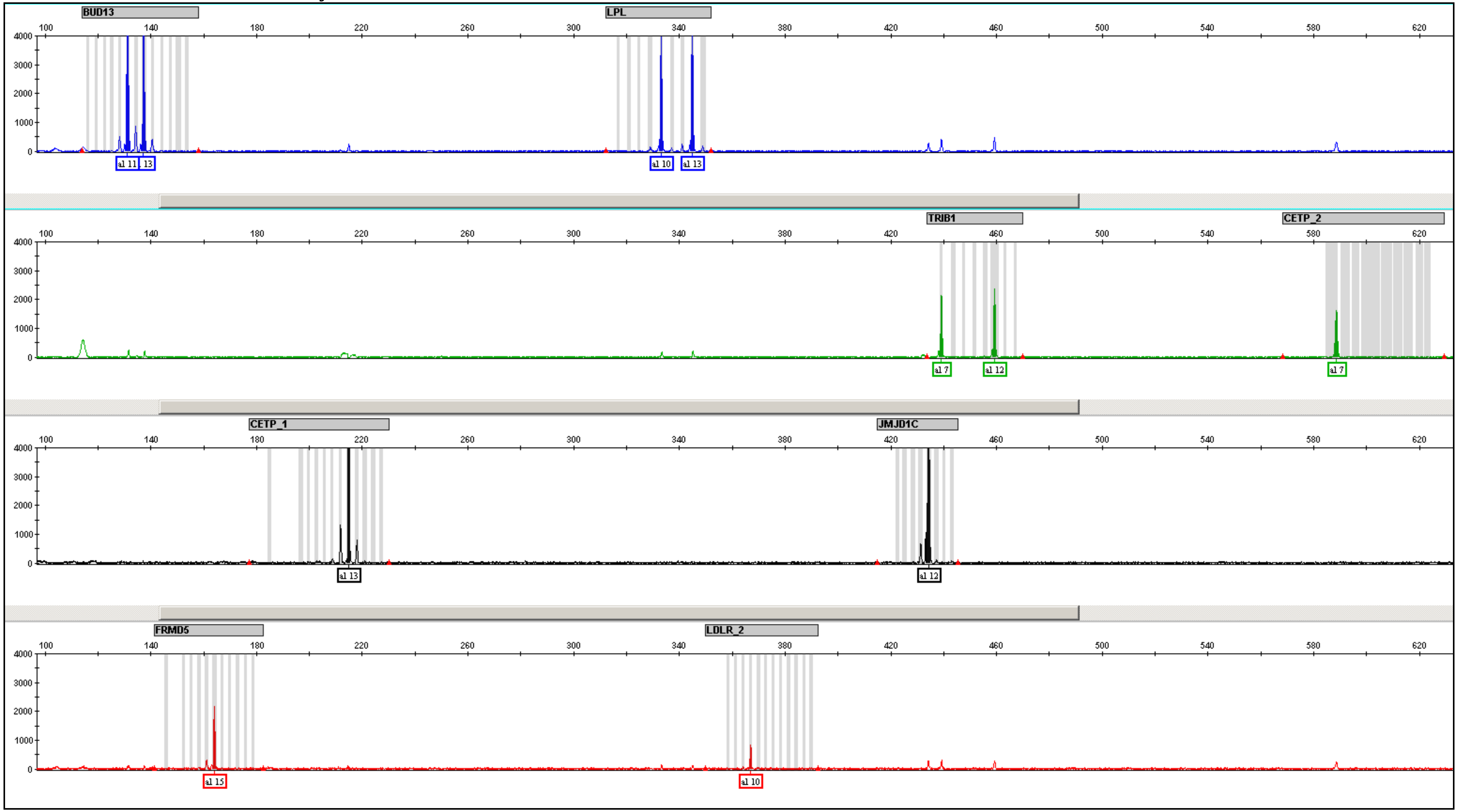

Figure S1b: Multiplex PCR amplification of the STRs: Electropherogram of the multiplex PCR 2 containing 8 STR loci after the electrophoresis on the ABI 3700s Genetic Analyzer.

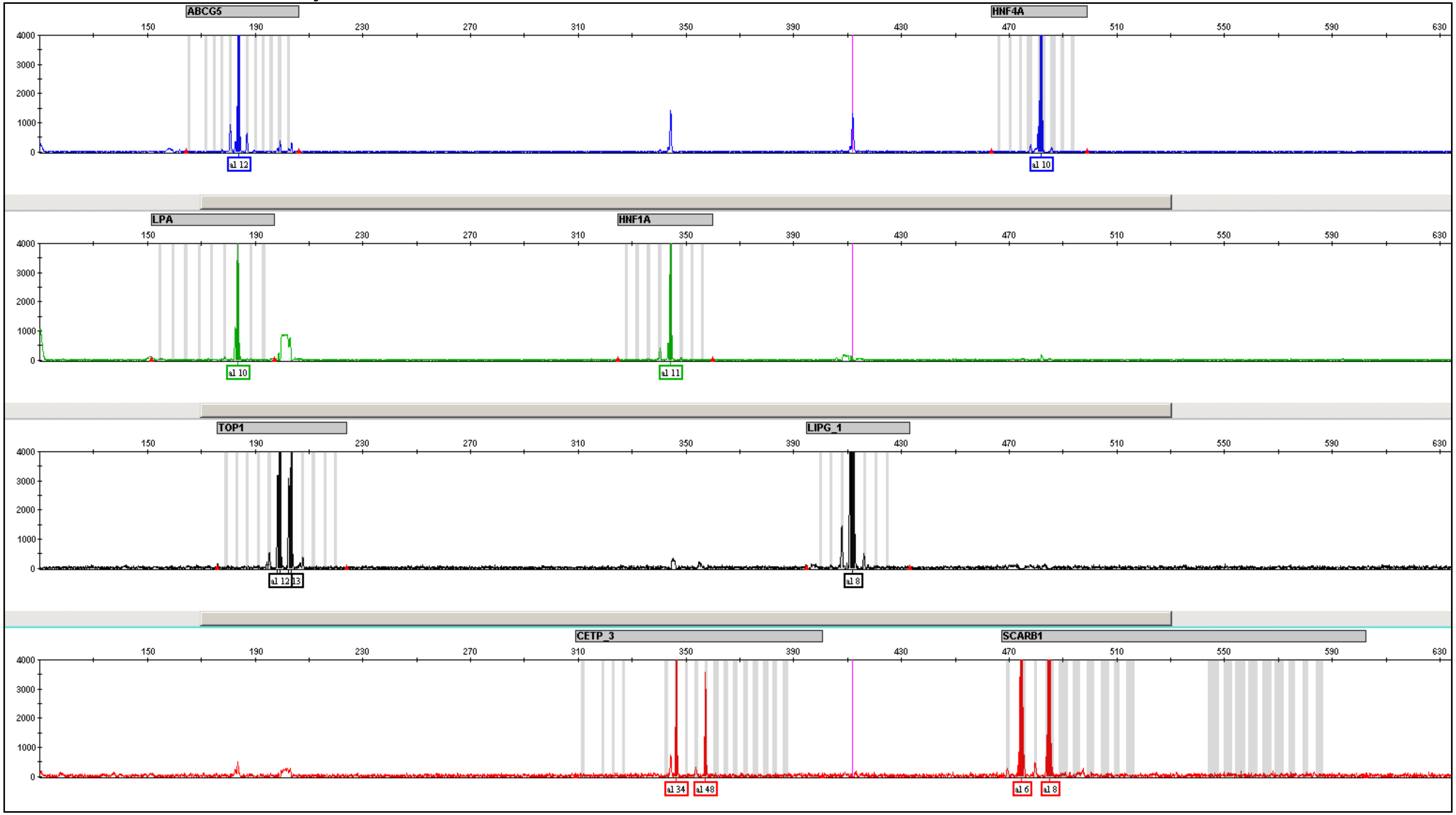

Figure S2: Distribution of number of repeats based on alleles and sum of alleles for each STR

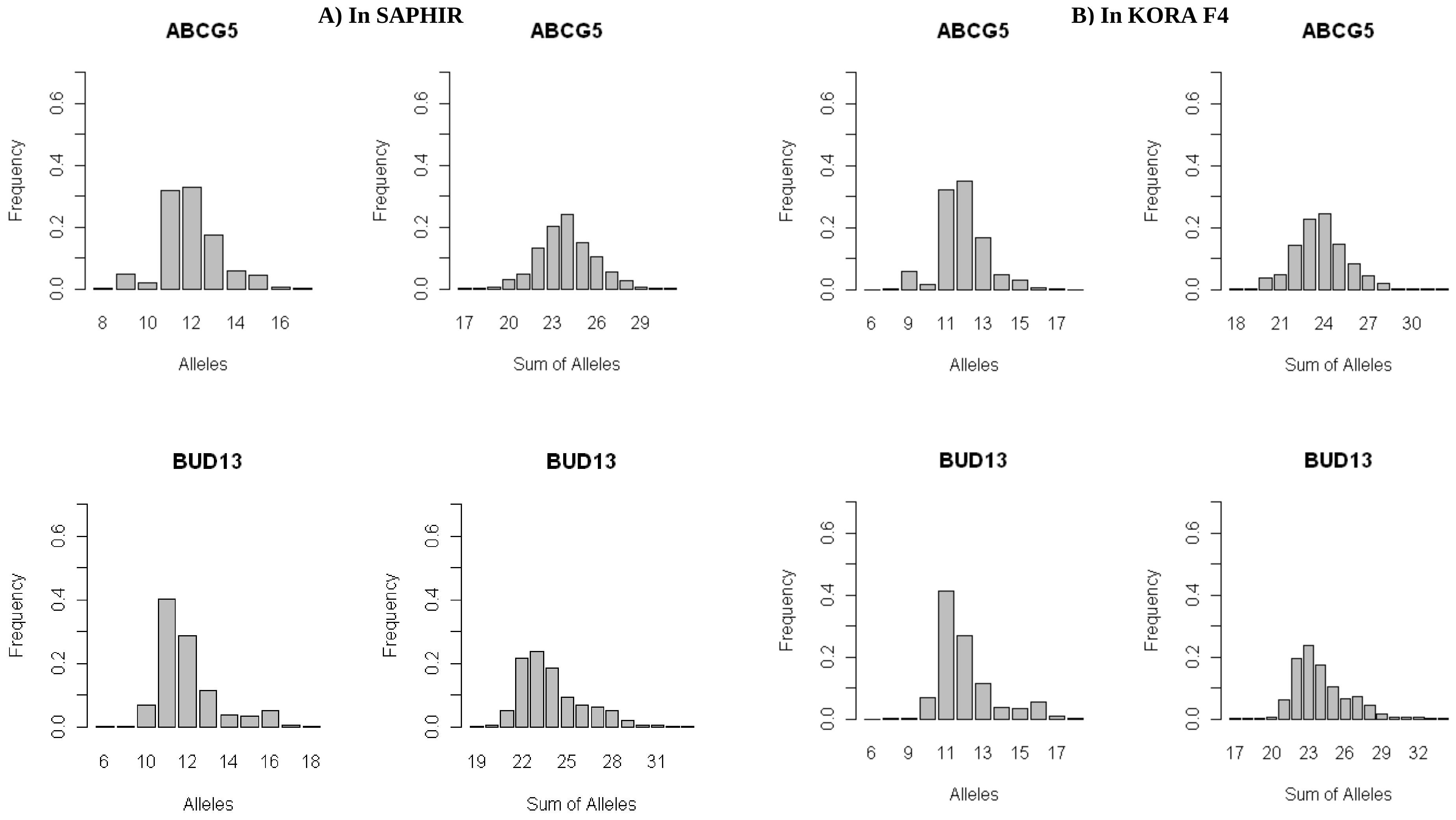

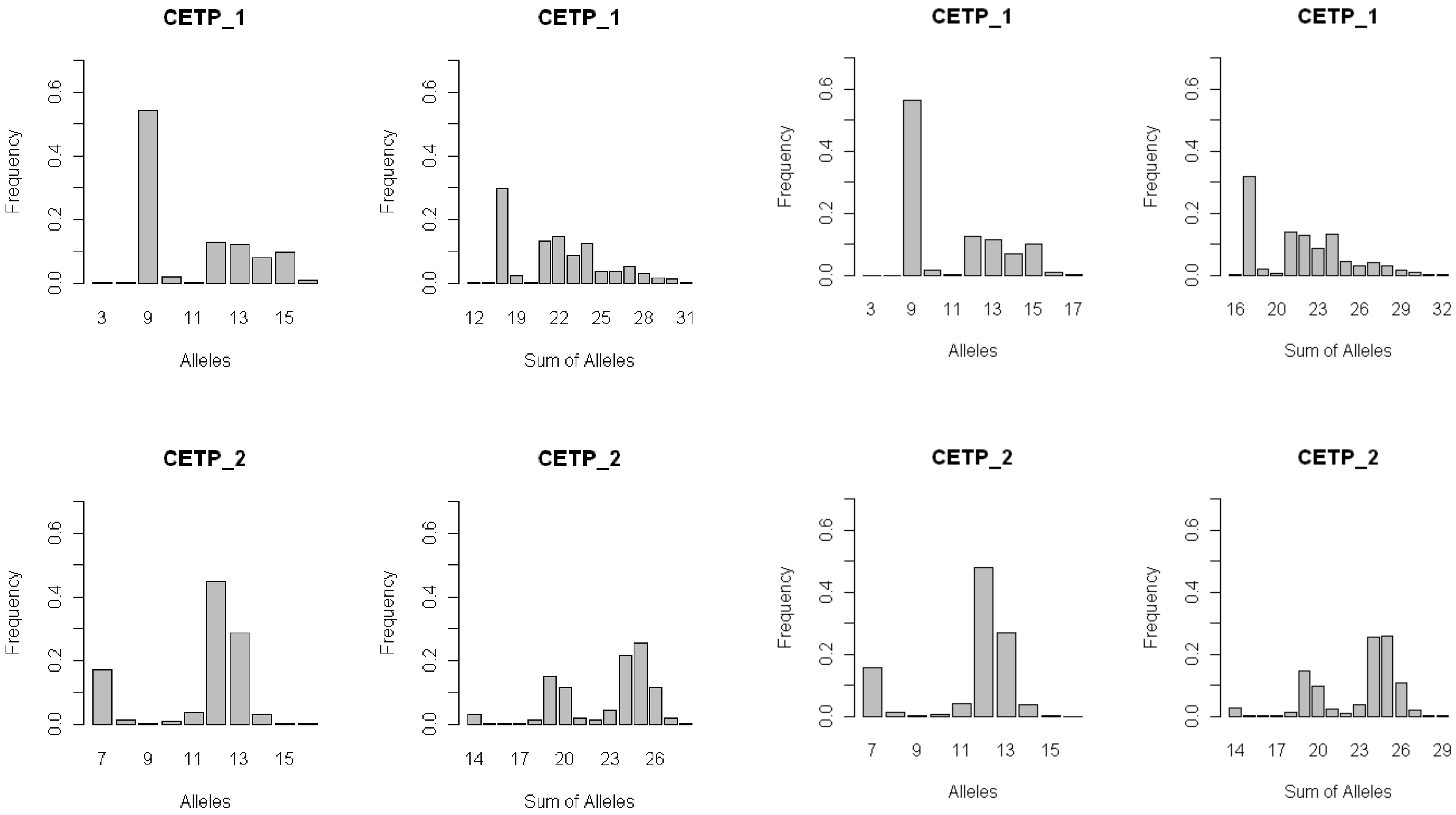
CETP_1
Frequency
0.0
0.2
0.4
0.6
3 9 11 13 15
Alleles
CETP_1
Frequency
0.0
0.2
0.4
0.6
12 19 22 25 28 31
Sum of Alleles
CETP_1
Frequency
0.0
0.2
0.4
0.6
3 9 11 13 15 17
Alleles
CETP_1
Frequency
0.0
0.2
0.4
0.6
16 20 23 26 29 32
Sum of Alleles
CETP_2
Frequency
0.0
0.2
0.4
0.6
7 9 11 13 15
Alleles
CETP_2
Frequency
0.0
0.2
0.4
0.6
14 17 20 23 26
Sum of Alleles
CETP_2
Frequency
0.0
0.2
0.4
0.6
7 9 11 13 15
Alleles
CETP_2
Frequency
0.0
0.2
0.4
0.6
14 17 20 23 26 29
Sum of Alleles

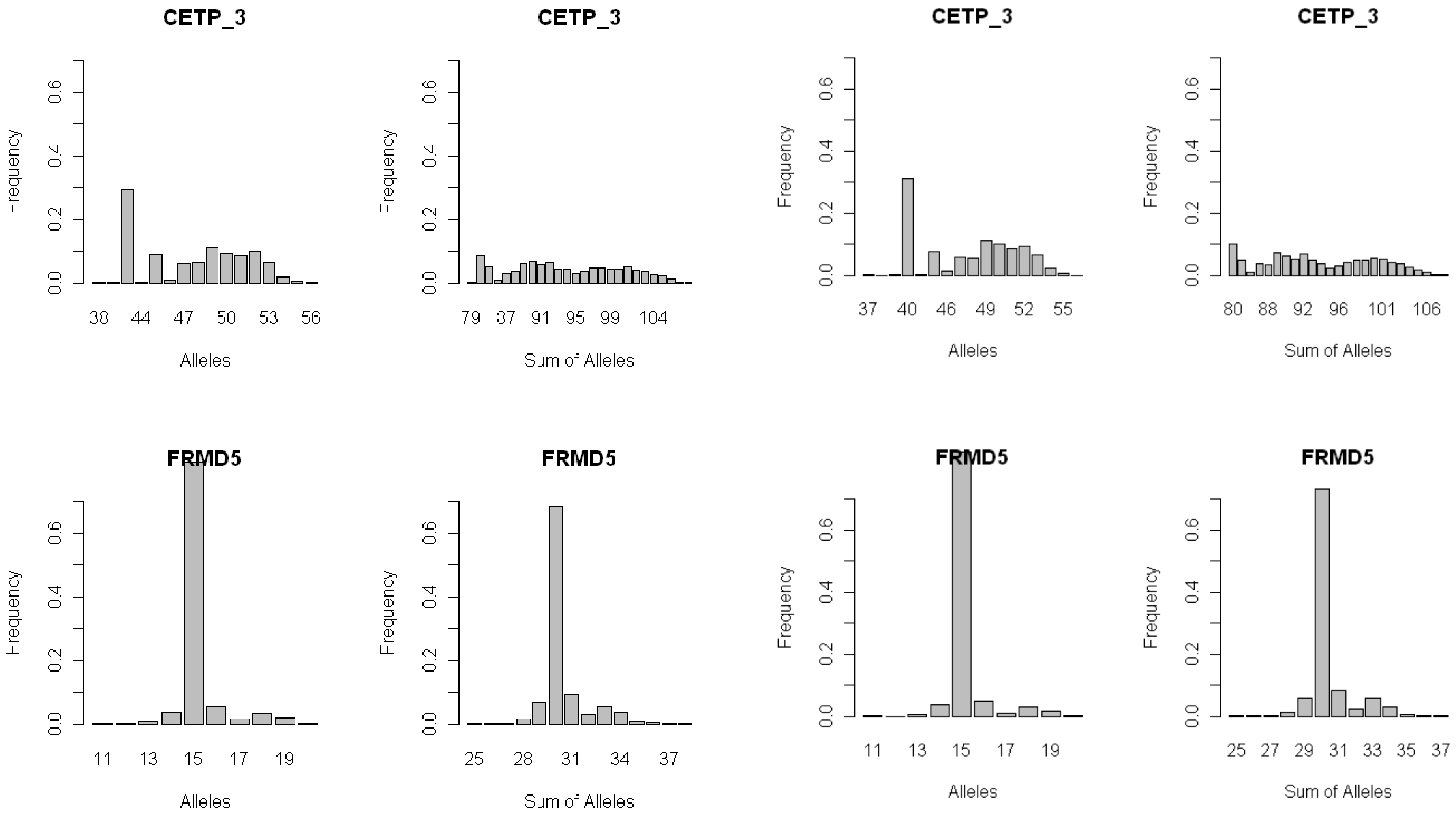
CETP_3
Frequency
0.0 0.2 0.4 0.6
38 44 47 50 53 56
Alleles
CETP_3
Frequency
0.0 0.2 0.4 0.6
79 87 91 95 99 104
Sum of Alleles
CETP_3
Frequency
0.0 0.2 0.4 0.6
37 40 46 49 52 55
Alleles
CETP_3
Frequency
0.0 0.2 0.4 0.6
80 88 92 96 101 106
Sum of Alleles
FRMD5
Frequency
0.0 0.2 0.4 0.6
11 13 15 17 19
Alleles
FRMD5
Frequency
0.0 0.2 0.4 0.6
25 28 31 34 37
Sum of Alleles
FRMD5
Frequency
0.0 0.2 0.4 0.6
11 13 15 17 19
Alleles
FRMD5
Frequency
0.0 0.2 0.4 0.6
25 27 29 31 33 35 37
Sum of Alleles

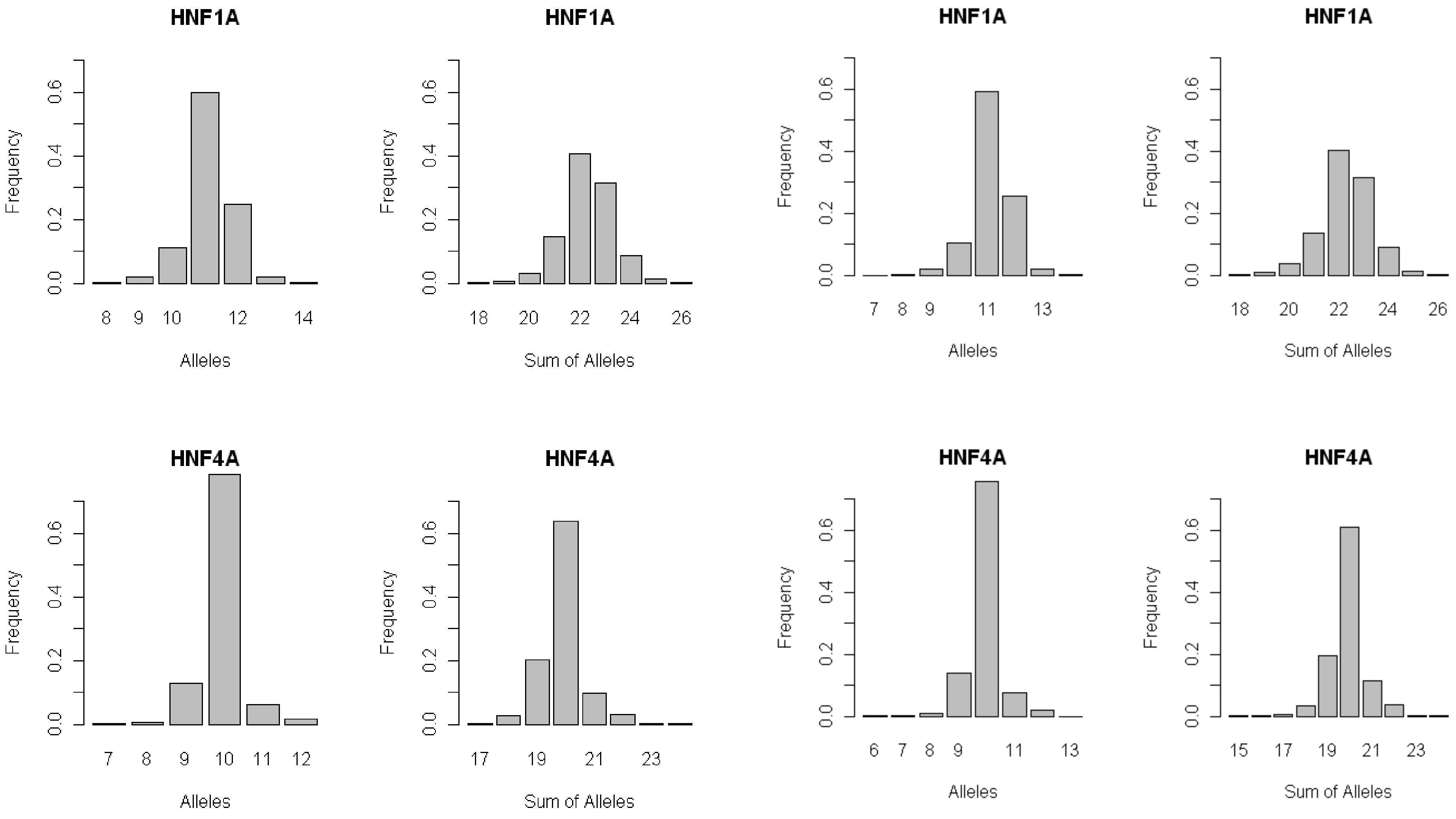
HNF1A
Frequency
Alleles
Sum of Alleles
HNF4A
0.0 0.2 0.4 0.6
8 9 10 12 14
18 20 22 24 26
7 8 9 11 13
7 8 9 10 11 12
17 19 21 23
6 7 8 9 11 13
15 17 19 21 23

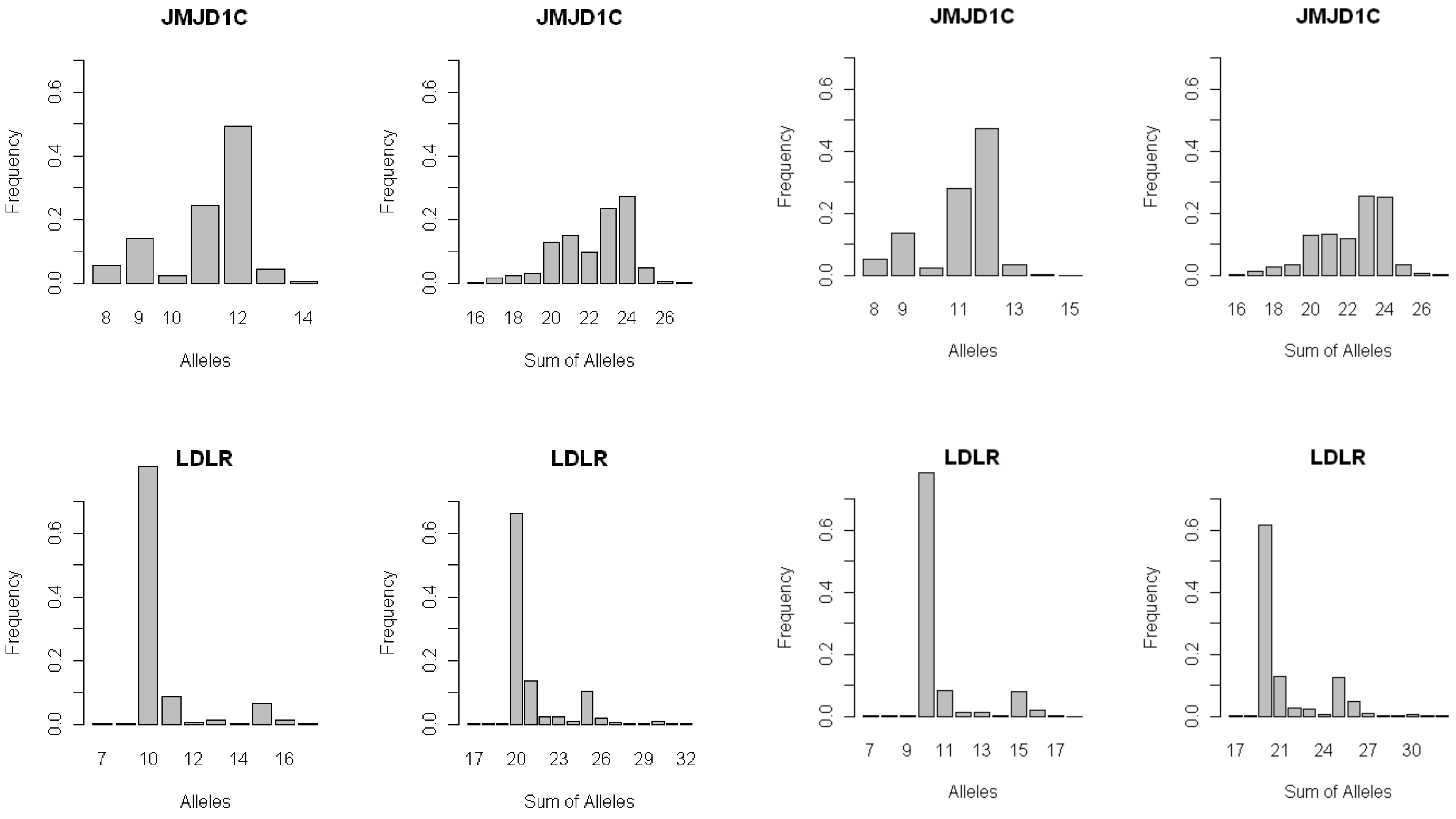
JMJD1C
Frequency
Alleles
Sum of Alleles
LDLR

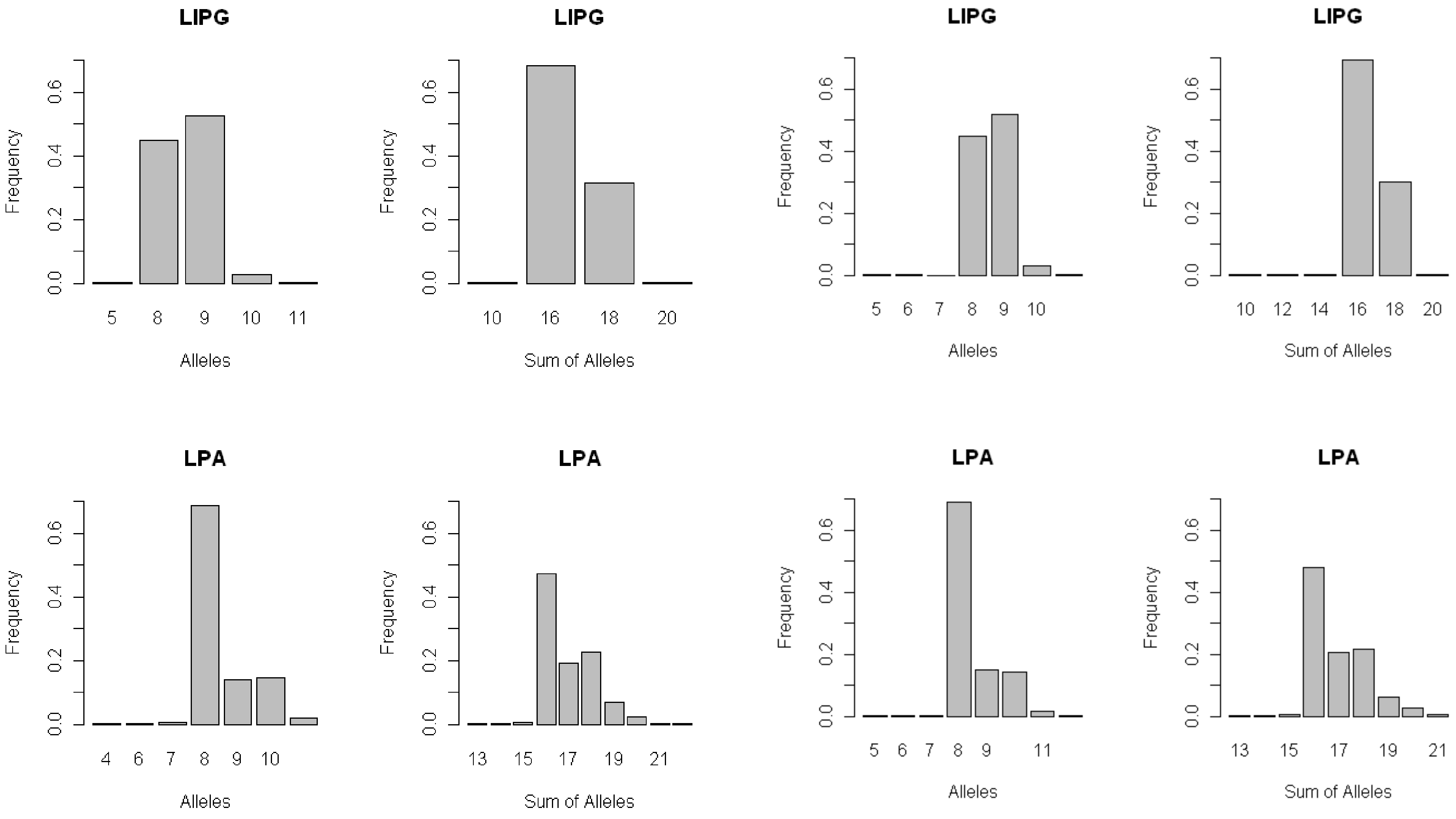
LIPG
Frequency
0.0
0.2
0.4
0.6
5
8
9
10
11
Alleles
LIPG
Frequency
0.0
0.2
0.4
0.6
10
16
18
20
Sum of Alleles
LIPG
Frequency
0.0
0.2
0.4
0.6
5
6
7
8
9
10
Alleles
LIPG
Frequency
0.0
0.2
0.4
0.6
10
12
14
16
18
20
Sum of Alleles
LPA
Frequency
0.0
0.2
0.4
0.6
4
6
7
8
9
10
Alleles
LPA
Frequency
0.0
0.2
0.4
0.6
13
15
17
19
21
Sum of Alleles
LPA
Frequency
0.0
0.2
0.4
0.6
5
6
7
8
9
11
Alleles
LPA
Frequency
0.0
0.2
0.4
0.6
13
15
17
19
21
Sum of Alleles

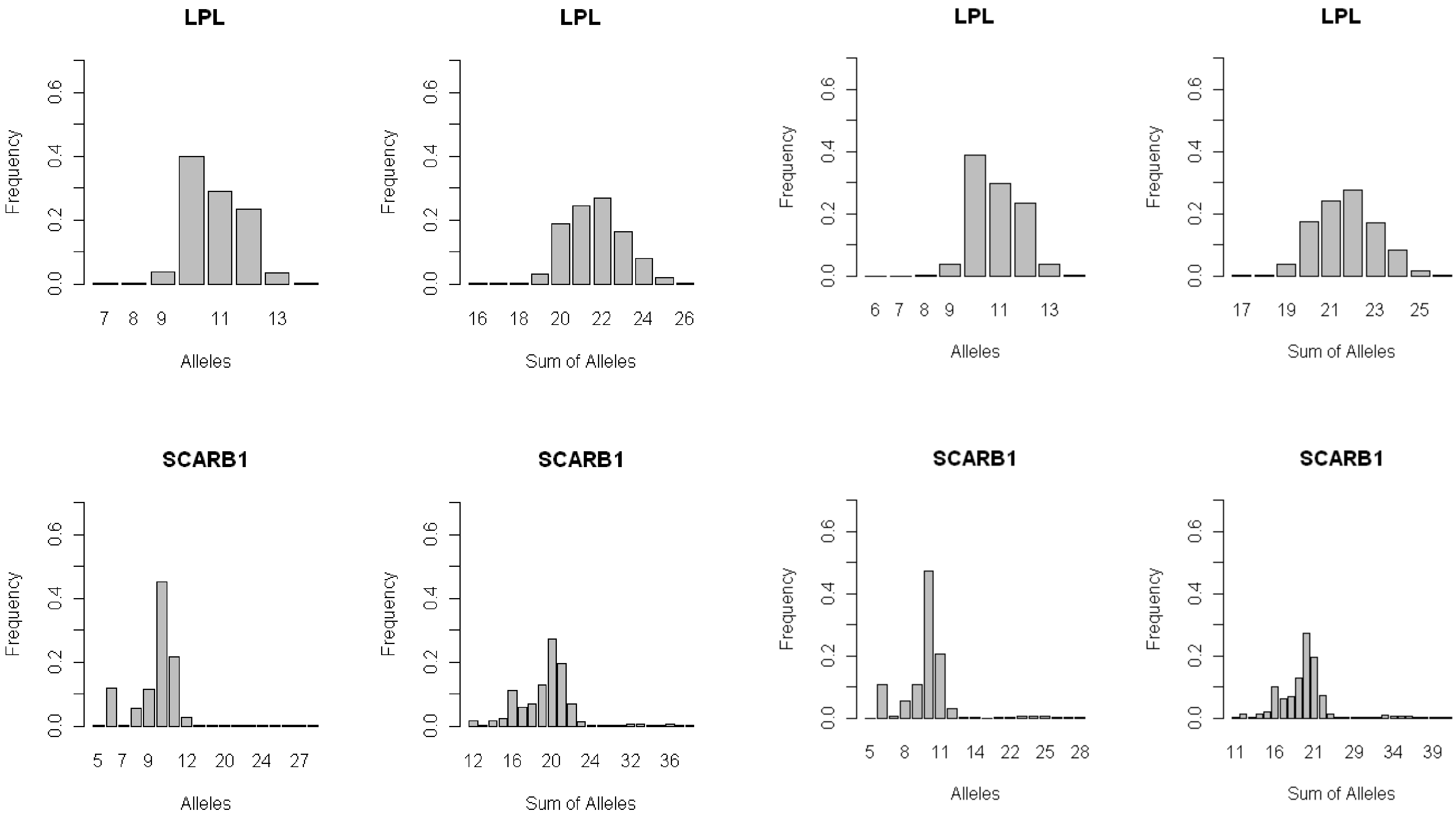
LPL
Frequency
0.0 0.2 0.4 0.6
7 8 9 11 13
Alleles
LPL
Frequency
0.0 0.2 0.4 0.6
16 18 20 22 24 26
Sum of Alleles
LPL
Frequency
0.0 0.2 0.4 0.6
6 7 8 9 11 13
Alleles
LPL
Frequency
0.0 0.2 0.4 0.6
17 19 21 23 25
Sum of Alleles
SCARB1
Frequency
0.0 0.2 0.4 0.6
5 7 9 12 20 24 27
Alleles
SCARB1
Frequency
0.0 0.2 0.4 0.6
12 16 20 24 32 36
Sum of Alleles
SCARB1
Frequency
0.0 0.2 0.4 0.6
5 8 11 14 22 25 28
Alleles
SCARB1
Frequency
0.0 0.2 0.4 0.6
11 16 21 29 34 39
Sum of Alleles

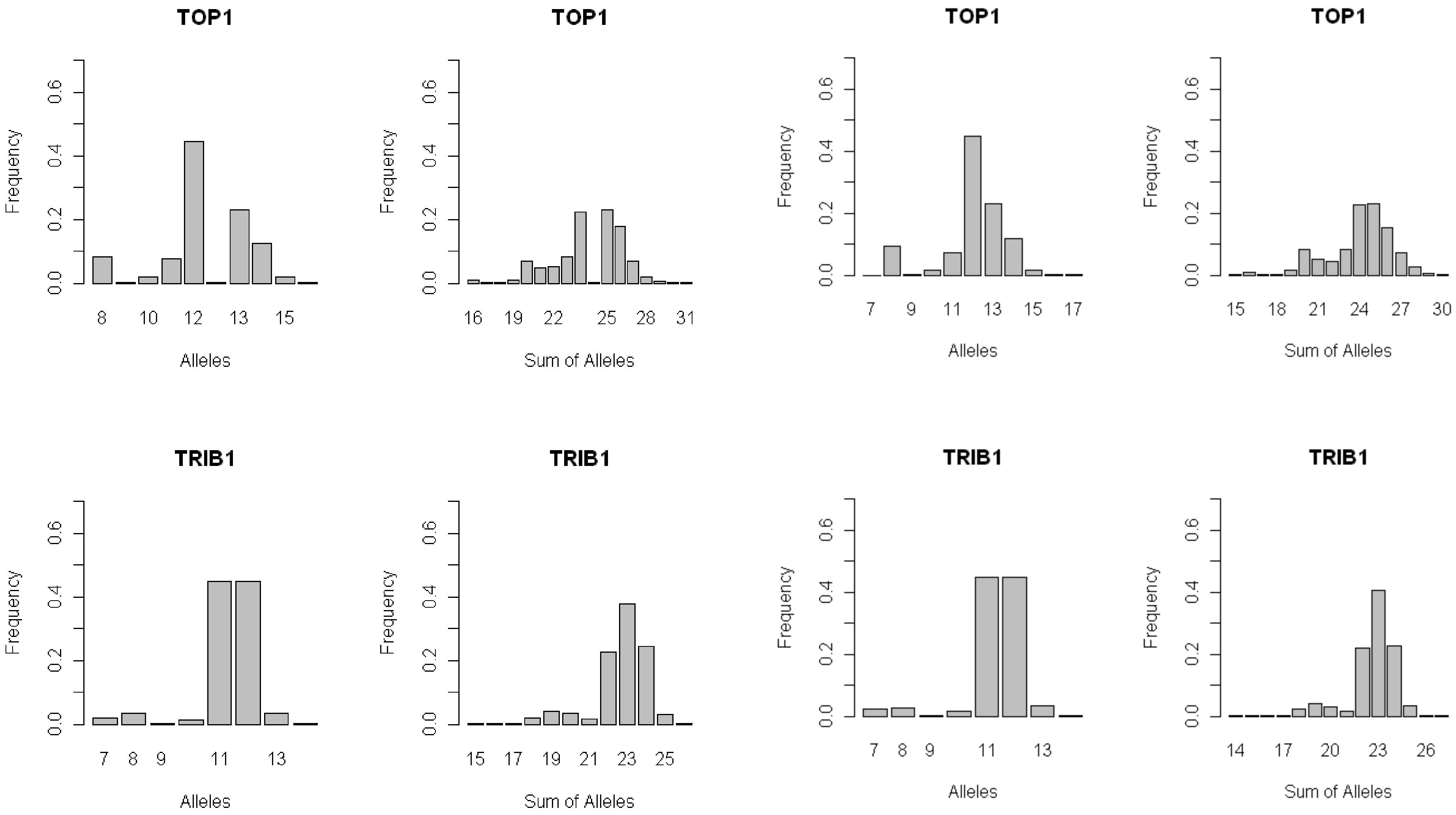
TOP1
Frequency
0.0 0.2 0.4 0.6
8 10 12 13 15
Alleles
TOP1
Frequency
0.0 0.2 0.4 0.6
16 19 22 25 28 31
Sum of Alleles
TOP1
Frequency
0.0 0.2 0.4 0.6
7 9 11 13 15 17
Alleles
TOP1
Frequency
0.0 0.2 0.4 0.6
15 18 21 24 27 30
Sum of Alleles
TRIB1
Frequency
0.0 0.2 0.4 0.6
7 8 9 11 13
Alleles
TRIB1
Frequency
0.0 0.2 0.4 0.6
15 17 19 21 23 25
Sum of Alleles
TRIB1
Frequency
0.0 0.2 0.4 0.6
7 8 9 11 13
Alleles
TRIB1
Frequency
0.0 0.2 0.4 0.6
14 17 20 23 26
Sum of Alleles

Table S2: Primer sequences, concentration and labelling for PCR amplification

8-plex PCR	Gene	Forward primer	Reverse primer	Final concentration in reaction mix [µM]	labelling
1	BUD13	*3´-TGAGAATGCCAGTTTGCCTA -5´*	*3´-GGCTACAATGGGGCATGATC -5´*	0.1	FAM
	LPL	*3´-AGGCCTTGGTGGGTGAATCA -5´*	*3´-CTGACCAAGGATAGTGGGATATAG -5´*	0.8	FAM
	CETP_1	*3´-AGAACTCTTTTCACCTTGAAAAACT -5´*	*3´-CCAGGAGTTTGAGGCTACAGT -5´*	0.8	AT550
	JMJD1C	*3´-CTTTTGGGGTACATTTATTCATGGATG -5´*	*3´-AGCTGAGAAACATATCAAGAAGGTG -5´*	0.2	AT550
	LDLR	*3´-AGACCTTTGTTGAGACAGAAAACC -5´*	*3´-TGATCAGTGCTTCTTTCCAAATAA -5´*	0.8	AT565
	FRMD5	*3´-CCCCAAAGAGTTTGAACATTGC -5´*	*3´-CTGAAGCATAGAAAGTGCTCAGTAA -5´*	0.2	AT565
	TRIB1	*3´-GCAGCTCAGTGGATCTTACATTC -5´*	*3´-AGTGTTGGGAAGGGCTTGAA -5´*	0.6	YY
	CETP_2	*3´-CGTCACCACATCCGCGTAAT -5´*	*3´-GGCTCCTGTTTCCGTGTTTG -5´*	0.8	YY
2	ABCG5	*3´-GGATAATGGCATGAACCTGGG -5´*	*3´-AGAAATCTACTGATGAGATGGCTTG -5´*	0.2	FAM
	HNF4A	*3´-CACTACACCCGACCAAGAATATTAA -5´*	*3´-TACAGATGGGCATGGGTTGCAT -5´*	0.2	FAM
	TOP1	*3´-CACTGGCCCTTAACTCTGCA -5´*	*3´-ATTTTCTTATATCACGATGTTACGC -5´*	0.4	AT550
	LIPG	*3´-CAGCCTGAGCAACATAGCGA -5´*	*3´-GCCTCCCAAGCTACTAGACTAC -5´*	0.6	AT550
	SCARB1	*3´-AATCGAAATGGACGTGTGAGG -5´*	*3´-GAAGGAATGAACAAATCGACACAG -5´*	0.2	AT565
	CETP_3	*3´-GCAAGAGAGTTGCTGGAACC -5´*	*3´-ATGGATTTGCCTACTCTGAAC -5´*	0.2	AT565
	LPA	*3´-CCACTTAATTCTACCACTCTTGCT -5´*	*3´-AGCCAAAATCACGTCAGTGC -5´*	0.2	YY
	HNF1A	*3´-ATTTGCATCATTTTATTCCTTGTCC -5´*	*3´-GGATGATGGATGGATGGACG -5´*	0.8	YY

Table S3a: Association of sum of alleles and single alleles in each STR with **Total Cholesterol** in SAPHIR, KORA and both studies combined, all age and sex-adjusted, excluding those individuals with lipid-lowering drugs; STRs are sorted by their p-value in Teslovich et al. (Teslovich et al., 2010)

**STR**	**p-value in Teslo-vich**	**SAPHIR (n=1648)** Linear model results based on sum of alleles		**% Var explained**	**Linear model results based on alleles**		**KORA (n=2553)** Linear model results		**% Var explained**	**Linear model results based on alleles**		**Combined (n=4201)** Linear Mixed model results based on sum of alleles	
		**beta**	**p-value**		beta	p-value*	**beta**	**p-value**		beta	p-value*	**beta**	**p-value**
LDLR	10^{-97}	-1.1168	0.0232	0.18	-1.191	0.0139	-1.4730	6.27E-06	0.31	-1.4526	1.43E-06	-1.3585	6.23E-07
BUD13	10^{-57}	1.8036	1.05E-04	1.18	1.7979	2.02E-04	0.8646	0.0132	0.18	0.8562	0.0184	1.2125	1.42E-05
ABCG5	10^{-45}	1.9165	1.93E-04	0.93	1.9256	3.59E-04	0.8436	0.0417	0.28	0.8304	0.0429	1.2864	6.51E-05
TRIB1	10^{-36}	-0.8563	0.1952	0.14	-0.8427	0.1979	0.0222	0.9652	0.00	0.0224	0.9643	-0.3257	0.4202
LIPG	10^{-19}	1.0323	0.3042	0.14	1.0663	0.3820	-0.6427	0.4117	0.15	-0.669	0.4655	0.0348	0.9551
LPA	10^{-17}	0.0053	0.9951	0.05	0.0051	0.9951	-0.2967	0.6539	0.07	-0.3006	0.6524	-0.1803	0.7313
TOP1	10^{-17}	0.1255	0.7768	0.03	0.1276	0.7596	-0.2416	0.4718	0.09	-0.2442	0.4736	-0.1001	0.7085
CETP_1	10^{-14}	0.2411	0.4178	0.13	0.2478	0.4119	0.4092	0.0787	0.07	0.4107	0.0844	0.3422	0.0620
CETP_2	10^{-14}	0.4891	0.1385	0.20	0.4863	0.1343	0.0401	0.8779	0.00	0.0401	0.8789	0.2187	0.2850
CETP_3	10^{-14}	-0.0255	0.8597	0.33	-0.0257	0.8553	-0.1176	0.2714	0.13	-0.1218	0.2686	-0.0822	0.3391
HNF1A	10^{-14}	-0.0653	0.9463	0.09	-0.064	0.9438	0.3866	0.6042	0.09	0.3802	0.6032	0.2143	0.7167
HNF4A	10^{-13}	-0.9796	0.4610	0.33	-0.9481	0.4614	0.3319	0.7186	0.00	0.3308	0.7364	-0.122	0.8723

*derived from a t-statistic based on robust standard error estimates using a sandwich variance-covariance-matrix

Table S3b: Association of sum of alleles and single alleles in each STR with **HDL Cholesterol** in SAPHIR, KORA and both studies combined, all age and sex-adjusted, excluding those individuals with lipid-lowering drugs; STRs are sorted by their p-value in Teslovich et al. (Teslovich et al., 2010)

**STR**	**p-value in Teslo-vich**	**SAPHIR (n=1648) Linear model results based on sum of alleles**		**% Var explained**	**Linear model results based on alleles**		**KORA (n=2553) Linear model results**		**% Var explained**	**Linear model results based on alleles**		**Combined (n=4201) Linear Mixed model results based on sum of alleles**	
		**beta**	**p-value**		beta	p-value*	**beta**	**p-value**		beta	p-value*	**beta**	**p-value**
CETP_1	10^{-380}	-0.3773	5.59E-04	0.74	-0.3878	3.32E-04	-0.5014	1.15E-09	1.23	-0.503	2.59E-10	-0.4513	7.62E-12
CETP_2	10^{-380}	0.5267	1.14E-05	1.1	0.5237	5.71E-06	0.5798	3.37E-10	1.14	0.5807	1.73E-11	0.5595	2.19E-14
CETP_3	10^{-380}	-0.3451	4.36E-11	2.58	-0.3482	3.11E-10	-0.3359	3.33E-19	2.88	-0.3477	1.01E-17	-0.3393	1.35E-28
LPL	10^{-98}	0.7685	0.0027	0.89	0.7973	0.0023	0.6140	0.0015	0.40	0.624	0.0027	0.6805	1.08E-05
LIPG	10^{-49}	-0.2485	0.5019	0.13	-0.1464	0.7585	-0.0799	0.7722	0.46	-0.0621	0.8540	-0.1516	0.4943
BUD13	10^{-47}	-0.7041	3.60E-05	1.03	-0.7018	4.55E-05	-0.1887	0.1278	0.28	-0.1868	0.1458	-0.3824	1.43E-04
TRIB1	10^{-19}	0.0797	0.7417	0.12	0.0784	0.7709	-0.0001	0.9997	0.00	-0.0001	0.9997	0.0327	0.8217
HNF4A	10^{-15}	0.7919	0.1034	0.18	0.7664	0.0967	0.2068	0.5259	0.56	0.2061	0.5487	0.4086	0.1338
SCARB1	10^{-14}	-0.0421	0.6998	0.00	-0.0428	0.6966	-0.0267	0.7186	0.09	-0.0269	0.7377	-0.0318	0.6063
LPA	10^{-08}	0.6593	0.0369	0.48	0.6323	0.0410	0.1836	0.4338	0.14	0.1861	0.4320	0.3687	0.0509

*derived from a t-statistic based on robust standard error estimates using a sandwich variance-covariance-matrix

Table S3c: Association of sum of alleles and single alleles in each STR with **LDL Cholesterol** in SAPHIR, KORA and both studies combined, all age and sex-adjusted, excluding those individuals with lipid-lowering drugs; STRs are sorted by their p-value in Teslovich et al. (Teslovich et al., 2010)

**STR**	**p-value in Teslo-vich**	**SAPHIR (n=1648)** Linear model results based on sum of alleles		**% Var explained**	**Linear model results based on alleles**		**KORA (n=2553)** Linear model results		**% Var explained**	**Linear model results based on alleles**		**Combined (n=4201)** Linear Mixed model results based on sum of alleles	
		**beta**	**p-value**		beta	p-value*	**beta**	**p-value**		beta	p-value*	**beta**	**p-value**
LDLR	10^{-117}	-1.0408	0.0218	0.14	-1.1111	0.0116	-1.5796	4.79E-08	0.72	-1.5576	7.63E-09	-1.4080	9.96E-09
ABCG5	10^{-47}	1.6747	4.05E-04	0.79	1.6842	6.13E-04	0.8251	0.0246	0.42	0.8122	0.0256	1.1806	4.70E-05
TRIB1	10^{-29}	-0.8923	0.1435	0.14	-0.8775	0.1576	0.0241	0.9576	0.00	0.0243	0.9577	-0.3377	0.3542
BUD13	10^{-26}	1.5535	3.03E-04	0.89	1.5477	3.06E-04	0.3190	0.3026	0.03	0.3159	0.3004	0.7840	0.0019
TOP1	10^{-19}	-0.0052	0.9898	0.00	-0.0053	0.9891	0.0835	0.7793	0.19	0.0843	0.7818	0.0502	0.8354
LPA	10^{-17}	-0.5933	0.4548	0.04	-0.5688	0.4489	-0.3293	0.5745	0.16	-0.3337	0.5736	-0.4301	0.3631
HNF1A	10^{-15}	-0.4598	0.6070	0.00	-0.4506	0.5810	0.1903	0.7729	0.23	0.1873	0.7718	-0.0712	0.8935
CETP_1	10^{-13}	0.6395	0.0199	0.34	0.6561	0.0179	0.7400	3.36E-04	0.44	0.7424	4.21E-04	0.6998	2.32E-05
CETP_2	10^{-13}	-0.0251	0.9342	0.01	-0.025	0.9351	-0.3524	0.1276	0.00	-0.3529	0.1378	-0.2221	0.2286
CETP_3	10^{-13}	0.2694	0.0432	0.34	0.2717	0.0370	0.1625	0.0863	0.40	0.1682	0.0857	0.2027	0.0089

*derived from a t-statistic based on robust standard error estimates using a sandwich variance-covariance-matrix

Table S3d: Association of sum of alleles and single alleles in each STR with **ln-transformed Triglycerides** in SAPHIR, KORA and both studies combined, all age and sex-adjusted, excluding those individuals with lipid-lowering drugs; STRs are sorted by their p-value in Teslovich et al. (Teslovich et al., 2010)

**STR**	**p-value in Teslo-vich**	**SAPHIR (n=1648)** **Linear model results based on sum of alleles**		**% Var explained**	**Linear model results based on alleles**		**KORA (n=2553)** **Linear model results**		**% Var explained**	**Linear model results based on alleles**		**Combined (n=4201)** **Linear Mixed model results based on sum of alleles**	
		**beta**	**p-value**		beta	p-value*	**beta**	**p-value**		beta	p-value*	**beta**	**p-value**
BUD13	10^{-240}	0.0408	2.46E-10	2.37	0.0407	7.19E-10	0.0252	2.02E-07	1.08	0.025	1.67E-06	0.0311	1.04E-15
LPL	10^{-115}	-0.0272	0.0052	0.42	-0.0282	0.0048	-0.0167	0.0285	0.08	-0.017	0.0286	-0.0208	5.34E-04
TRIB1	10^{-55}	0.0012	0.8934	0.00	0.0012	0.8955	0.0022	0.7547	0.00	0.0022	0.7559	0.0019	0.7397
CETP_1	10^{-12}	0.0015	0.7252	0.10	0.0015	0.7272	0.0082	0.0112	0.26	0.0083	0.0107	0.0056	0.0295
CETP_2	10^{-12}	-0.0049	0.2838	0.06	-0.0048	0.2879	-0.0087	0.0168	0.25	-0.0087	0.0168	-0.0071	0.0120
CETP_3	10^{-12}	0.0027	0.1787	0.11	0.0027	0.1764	0.0030	0.0410	0.33	0.0031	0.0378	0.0029	0.0146
JMJD1C	10^{-12}	-0.0042	0.5688	0.04	-0.0041	0.5626	-0.0114	0.0610	0.18	-0.0118	0.0670	-0.0085	0.0714
FRMD5	10^{-11}	0.015	0.1455	0.29	0.0157	0.1258	0.0104	0.2507	0.02	0.01	0.2649	0.0125	0.0647

*derived from a t-statistic based on robust standard error estimates using a sandwich variance-covariance-matrix

Table S4a: Regression on **Total cholesterol** in KORA F4: Comparing the results for the sum of alleles of STRs, for the lead SNP within that gene region (= best SNP in Teslovich et al.) and for the sum of alleles of STRs, adjusted for the lead SNP (this latter analysis only given for STRs, which were significantly associated with lipids in the combined analysis in SAPHIR & KORA F4). All regression models were adjusted for age and sex, excluding those individuals with lipid-lowering drugs; STRs are sorted by their p-value in Teslovich et al. (Teslovich et al., 2010)

STR	p-value in Teslovich	STR (sum of alleles) Linear model results		Lead SNP from Teslovich et al. Linear model results			r^2 between lead SNP and STR	STR, adjusted for lead SNP Linear model results	
		beta	p-value	rs-number	beta[a]	p-value		beta	p-value
**LDLR**	10^{-97}	-1.4730	6.27E-06	rs6511720	-8.0470	2.01E-06	0.8660	-0.4522	0.6111
**BUD13**	10^{-57}	0.8646	0.0132	rs964184	5.4035	3.56E-04	0.5668	-0.0798	0.8800
**ABCG5**	10^{-45}	0.8436	0.0417	rs4299376	2.5913	0.0234	0.1556	0.5433	0.2277
TRIB1	10^{-36}	0.0222	0.9652	rs2954029	-3.8661	2.8E-04			
LIPG	10^{-19}	-0.6427	0.4117	rs7241918	-0.4788	0.7454			
LPA	10^{-17}	-0.2967	0.6539	rs1564348	1.2628	0.3754			
TOP1	10^{-17}	-0.2416	0.4718	rs6029526	-0.1515	0.8867			
CETP_1	10^{-14}	0.4092	0.0787	rs3764261	1.3793	0.2220			
CETP_2	10^{-14}	0.0401	0.8779	rs3764261	1.3793	0.2220			
CETP_3	10^{-14}	-0.1176	0.2714	rs3764261	1.3793	0.2220			
HNF1A	10^{-14}	0.3866	0.6042	rs1169288	0.6727	0.5677			
HNF4A	10^{-13}	0.3319	0.7186	rs1800961	-10.8245	0.0012			

[a] beta effect: estimate for the lead SNP refers to the minor allele, assuming an additive model.

Table S4b: Regression on **HDL cholesterol** in KORA F4: Comparing the results for the sum of alleles of STRs, for the lead SNP within that gene region (= best SNP in Teslovich et al.) and for the sum of alleles of STRs, adjusted for the lead SNP (this latter analysis only given for STRs, which were significantly associated with lipids in the combined analysis in SAPHIR & KORA F4). All regression models were adjusted for age and sex, excluding those individuals with lipid-lowering drugs; STRs are sorted by their p-value in Teslovich et al. (Teslovich et al., 2010)

STR	p-value in Teslovich	STR (sum of alleles) Linear model results		Lead SNP from Teslovich et al. Linear model results			r^2 between lead SNP and STR	STR, adjusted for lead SNP Linear model results	
		beta	p-value	rs-number	beta[a]	p-value		beta	p-value
**CETP_1**	10^{-380}	-0.5014	1.15E-09	rs3764261	4.0230	3.67E-24	0.3008	-0.0715	0.4610
**CETP_2**	10^{-380}	0.5798	3.37E-10	rs3764261	4.0230	3.67E-24	0.0463	0.4015	1.55e-05
**CETP_3**	10^{-380}	-0.3359	3.33E-19	rs3764261	4.0230	3.67E-24	0.8259	-0.0085	0.9240
**LPL**	10^{-98}	0.6140	0.0015	rs12678919	1.8011	0.0042	0.1609	0.4706	0.0259
LIPG	10^{-49}	-0.0799	0.7722	rs7241918	- 1.9718	1.58E-04			
**BUD13**	10^{-47}	-0.1887	0.1278	rs964184	- 1.2328	0.0216	0.5668	0.0275	0.884
TRIB1	10^{-19}	-0.0001	0.9997	rs2954029	0.5070	0.1800			
HNF4A	10^{-15}	0.2068	0.5259	rs1800961	-2.1401	0.0722			
SCARB1	10^{-14}	-0.0267	0.7186	rs838880	0.6316	0.2450			
LPA	10^{-08}	0.1836	0.4338	rs1084651	n.a.	n.a.			

[a] beta effect: estimate for the lead SNP refers to the minor allele, assuming an additive model.

Table S4c: Regression on **LDL cholesterol** in KORA F4: Comparing the results for the sum of alleles of STRs, for the lead SNP within that gene region (= best SNP in Teslovich et al.) and for the sum of alleles of STRs, adjusted for the lead SNP (this latter analysis only given for STRs, which were significantly associated with lipids in the combined analysis in SAPHIR & KORA F4). All regression models were adjusted for age and sex, excluding those individuals with lipid-lowering drugs; STRs are sorted by their p-value in Teslovich et al. (Teslovich et al., 2010)

STR	p-value in Teslovich	STR (sum of alleles) Linear model results		Lead SNP from Teslovich et al. Linear model results			r^2 between lead SNP and STR	STR, adjusted for lead SNP Linear model results	
		beta	p-value	rs-number	beta[a]	p-value		beta	p-value
**LDLR**	10^{-117}	-1.5796	4.79E-08	rs6511720	-8.689	7.01E-09	0.8660	-0.4325	0.5831
**ABCG5**	10^{-47}	0.8251	0.0246	rs4299376	1.8916	0.0622	0.0001	0.6331	0.1128
TRIB1	10^{-29}	0.0241	0.9576	rs2954029	-2.7966	0.0031			
BUD13	10^{-26}	0.3190	0.3026	rs964184	2.4573	0.0675			
TOP1	10^{-19}	0.0835	0.7793	rs6029526	0.6681	0.4788			
LPA	10^{-17}	-0.3293	0.5745	rs1564348	1.0277	0.4171			
HNF1A	10^{-15}	0.1903	0.7729	rs1169288	0.4144	0.6916			
**CETP_1**	10^{-13}	0.7400	3.36E-04	rs3764261	-2.2240	0.0265	0.3008	0.7265	0.0033
CETP_2	10^{-13}	-0.3524	0.1276	rs3764261	-2.2240	0.0265			
CETP_3	10^{-13}	0.1625	0.0863	rs3764261	-2.2240	0.0265			

[a] beta effect: estimate for the lead SNP refers to the minor allele, assuming an additive model.

Table S4d: Regression on **ln-transformed Triglycerides** in KORA F4: Comparing the results for the sum of alleles of STRs, for the lead SNP within that gene region (= best SNP in Teslovich et al.) and for the sum of alleles of STRs, adjusted for the lead SNP (this latter analysis only given for STRs, which were significantly associated with lipids in the combined analysis in SAPHIR & KORA F4). All regression models were adjusted for age and sex, excluding those individuals with lipid-lowering drugs; STRs are sorted by their p-value in Teslovich et al. (Teslovich et al., 2010)

**STR**	**p-value in Teslovich**	**STR (sum of alleles) Linear model results**		**Lead SNP from Teslovich et al. Linear model results**			**r^2 between lead SNP and STR**	**STR, adjusted for lead SNP Linear model results**	
		**beta**	**p-value**	**rs-number**	**beta[a]**	**p-value**		**beta**	**p-value**
**BUD13**	10^{-240}	0.0252	2.02E-07	rs964184	0.1532	3.16e-13	0.5668	-0.0011	0.8810
**LPL**	10^{-115}	-0.0167	0.0285	rs12678919	-0.0963	9.88e-05	0.1609	-0.0059	0.4772
TRIB1	10^{-55}	0.0022	0.7547	rs2954029	-0.0637	1.73e-05			
CETP_1	10^{-12}	0.0082	0.0112	rs3764261	-0.0347	0.0275			
CETP_2	10^{-12}	-0.0087	0.0168	rs3764261	-0.0347	0.0275			
CETP_3	10^{-12}	0.0030	0.0410	rs3764261	-0.0347	0.0275			
JMJD1C	10^{-12}	-0.0114	0.0610	rs10761731	-0.0517	4.88E-04			
FRMD5	10^{-11}	0.0104	0.2507	rs2929282	0.0562	0.1010			

[a] beta effect estimate for the lead SNP refers to the minor allele, assuming an additive model.

Figure S3a: Regional plots showing the association of SNPs and STRs in the *LDLR* gene region on Total Cholesterol

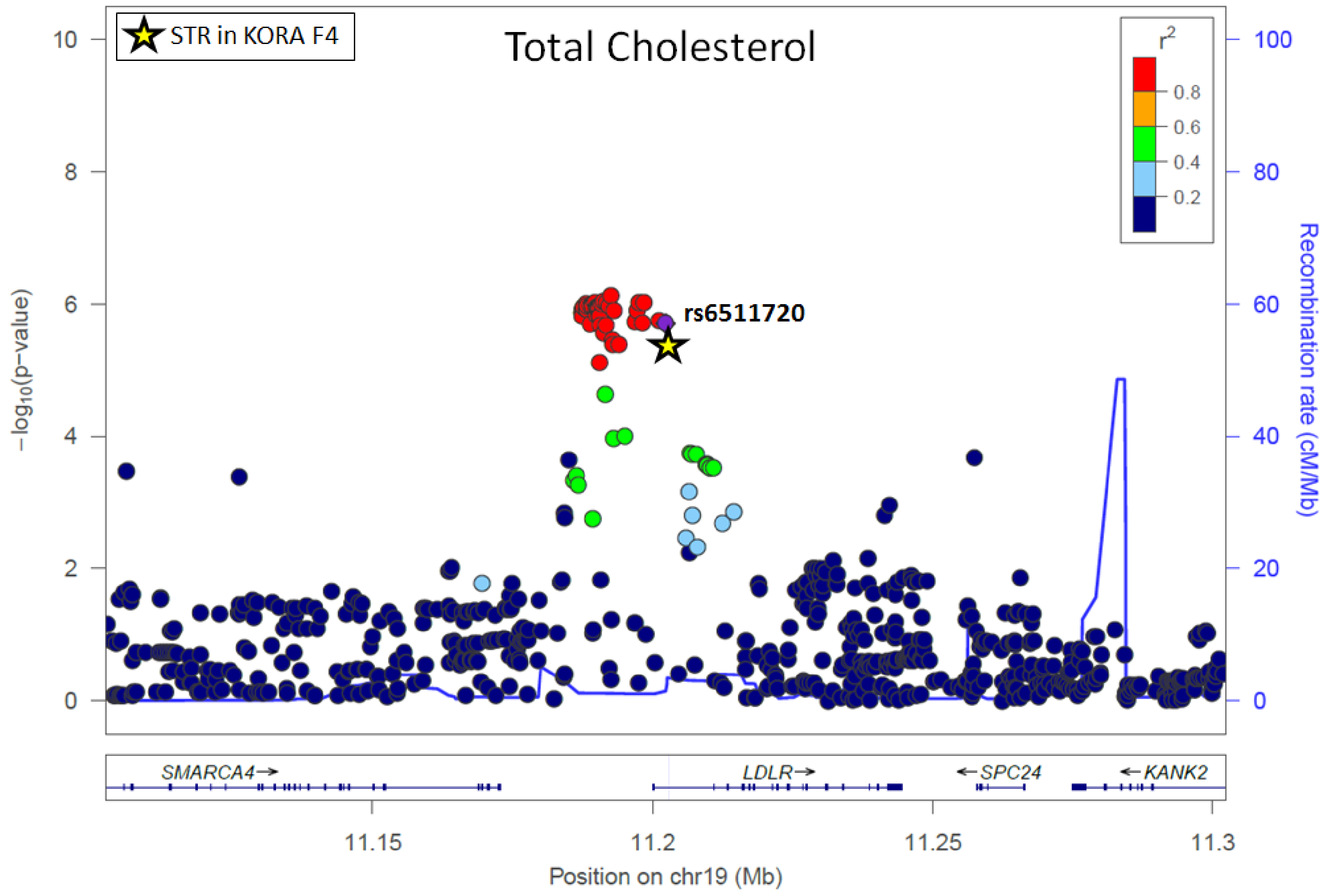

Figure S3b: Regional plots showing the association of SNPs and STRs in the *APOA1/C3/A4/A5/BUD13* gene region on Total Cholesterol

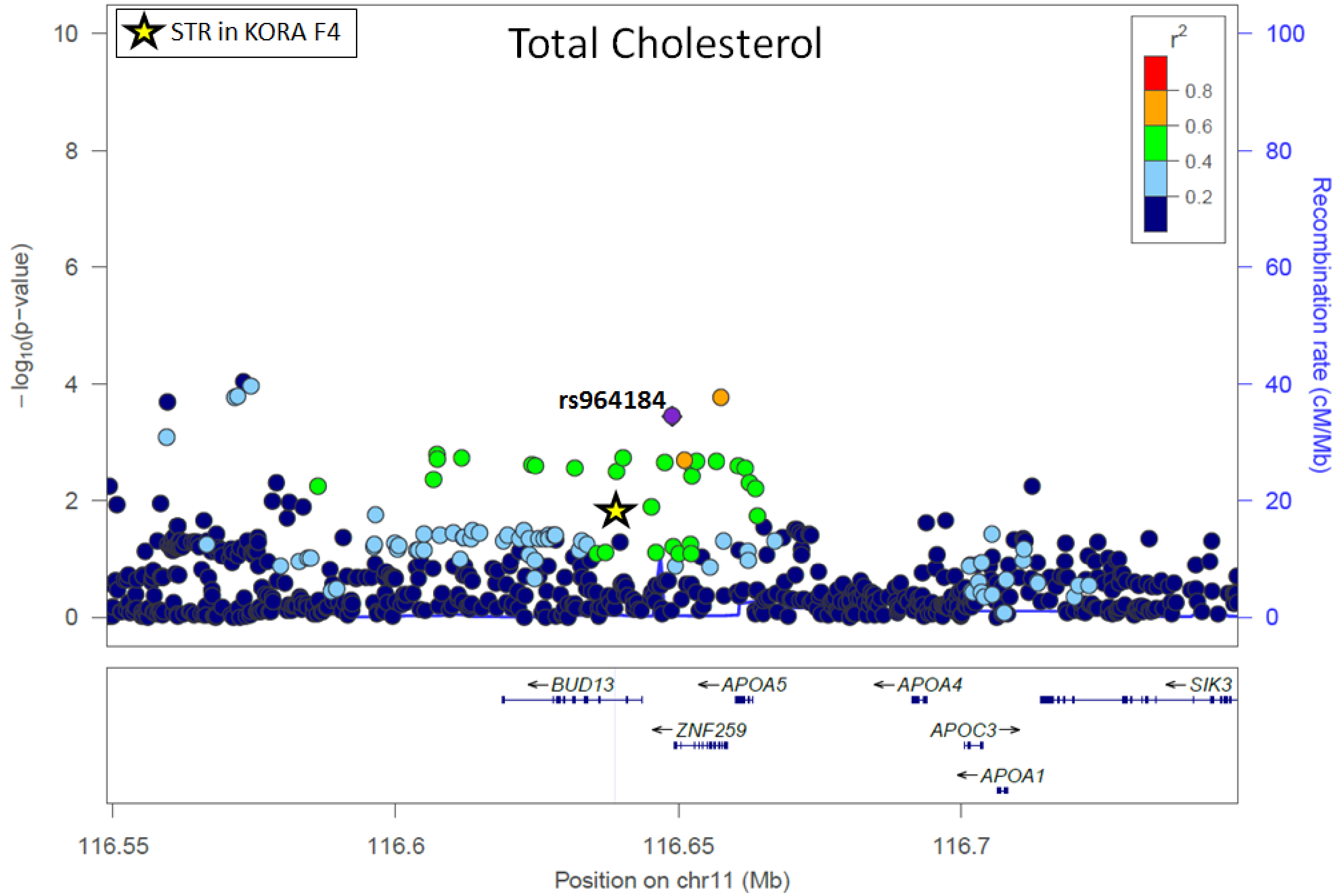

Figure S3c: Regional plots showing the association of SNPs and STRs in the *ABCG5/8* gene region on Total Cholesterol

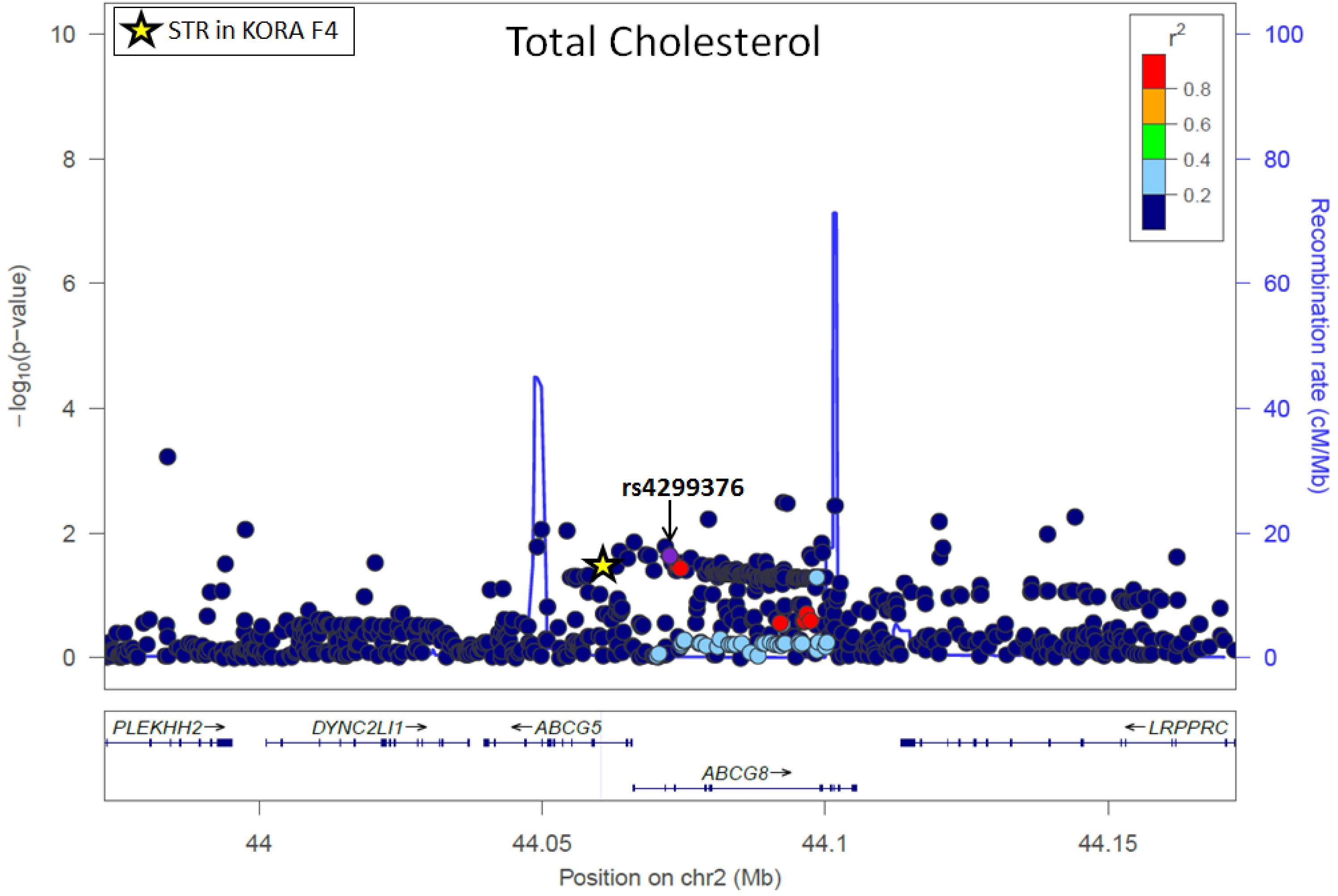

Figure S3d: Regional plots showing the association of SNPs and STRs in the *CETP* gene region on HDL Cholesterol

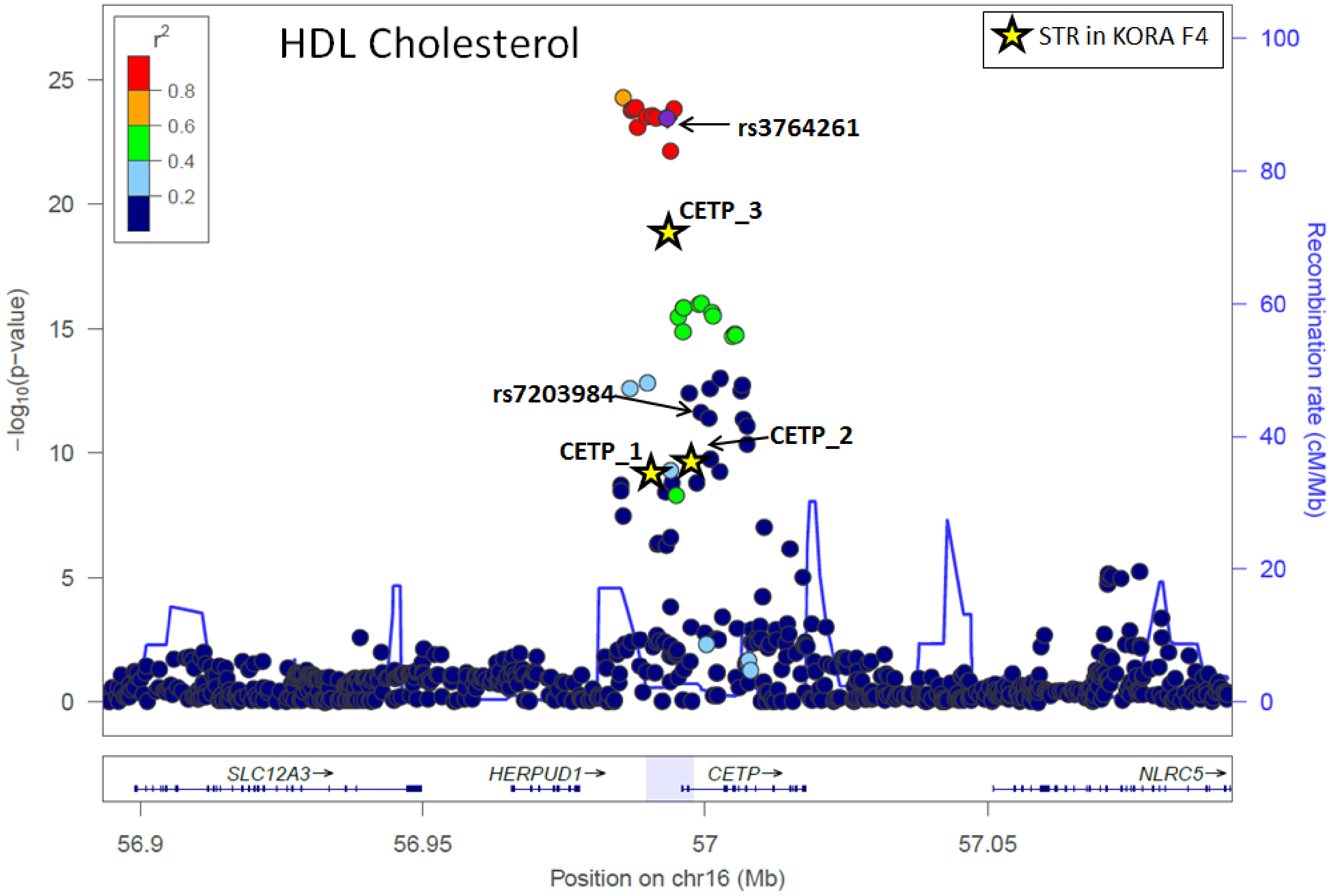

Figure S3e) Regional plots showing the association of SNPs and STRs in the *LPL* gene region on HDL Cholesterol

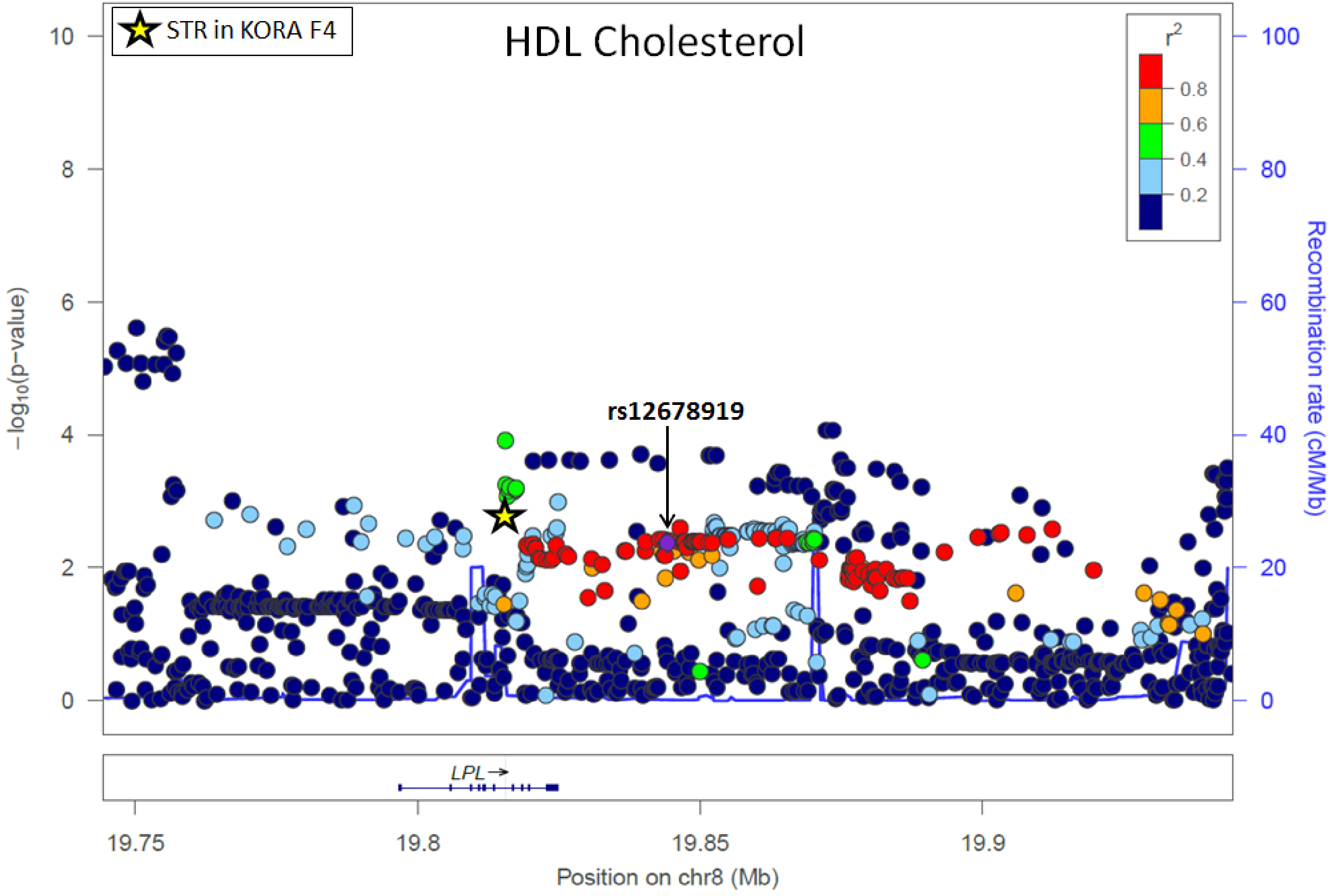

Figure S3f: Regional plots showing the association of SNPs and STRs in the *APOA1/C3/A4/A5/BUD13* gene region on HDL Cholesterol

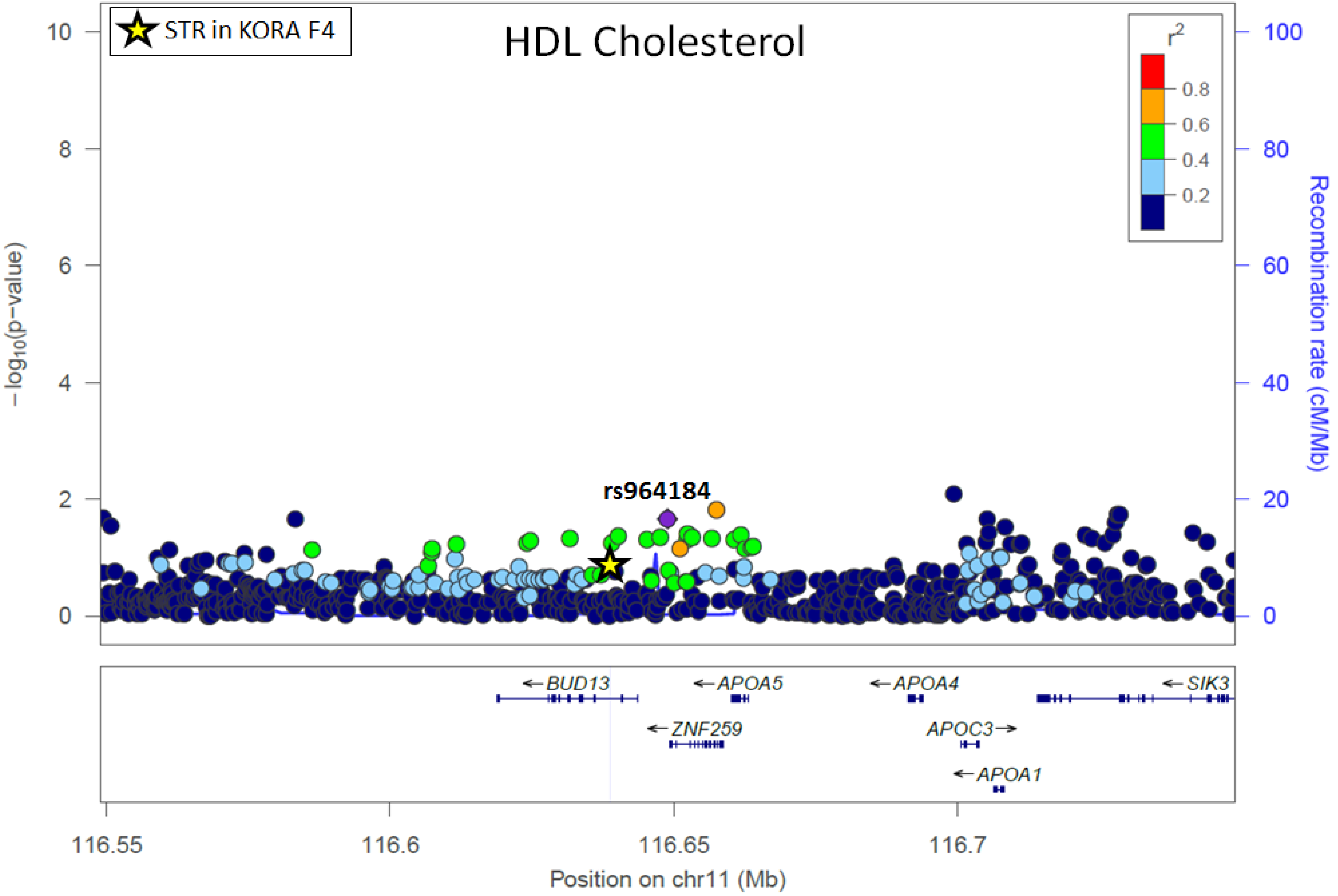

Figure S3g: Regional plots showing the association of SNPs and STRs in the *LDLR* gene region on HDL Cholesterol

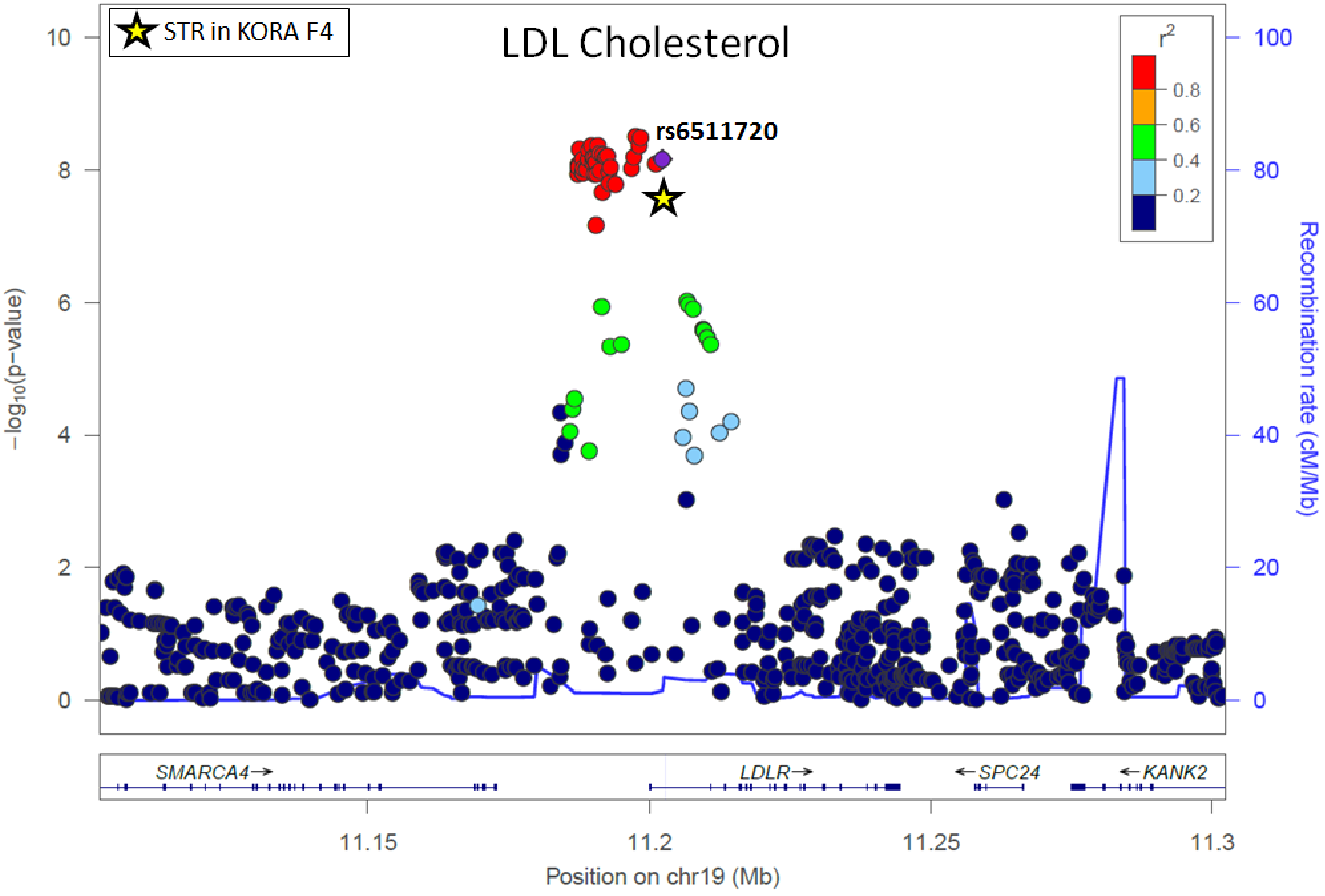

Figure S3h: Regional plots showing the association of SNPs and STRs in the *ABCG5/8* gene region on LDL Cholesterol

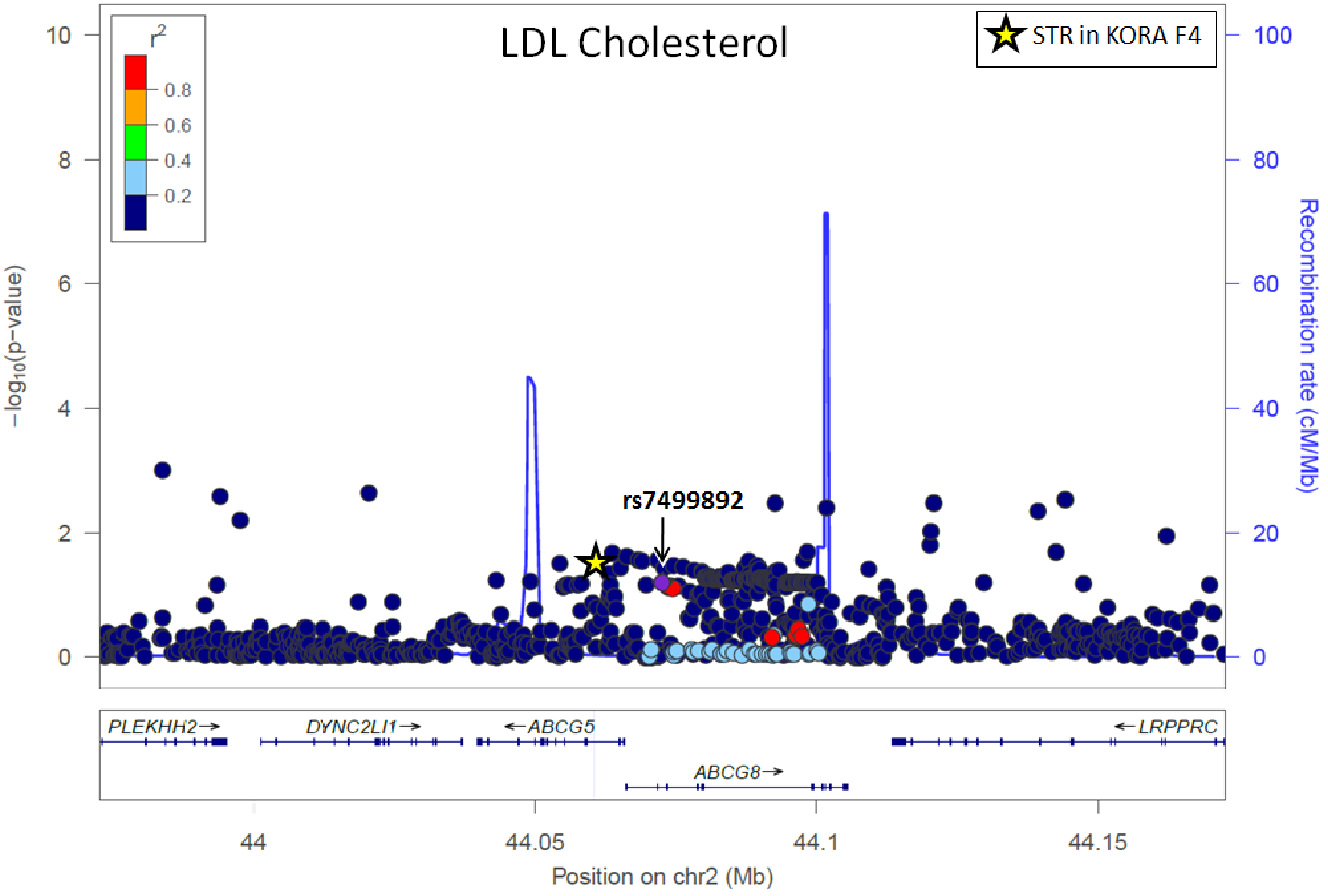

Figure S3i: Regional plots showing the association of SNPs and STRs in the *CETP* gene region on LDL Cholesterol

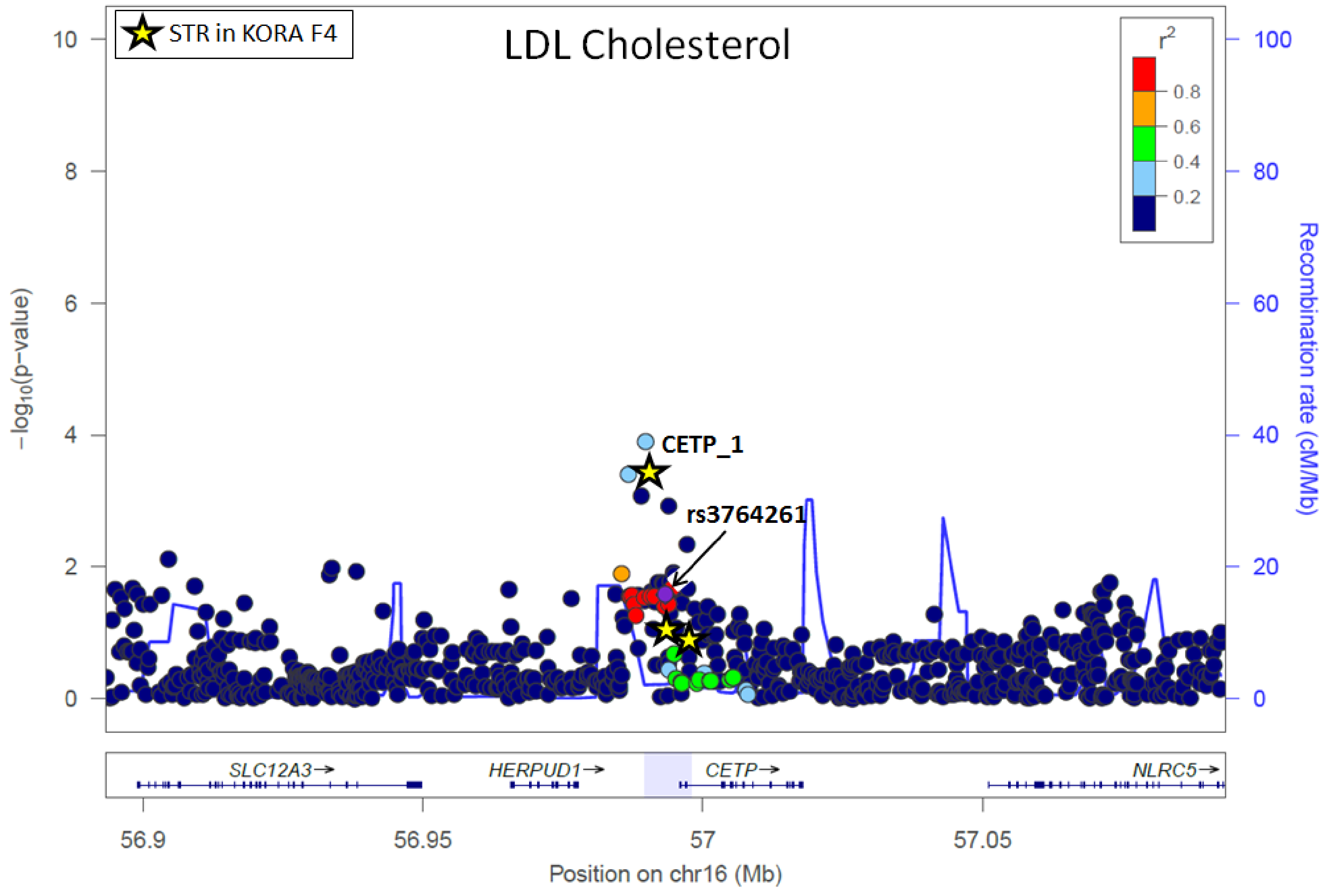

Figure S3j: Regional plots showing the association of SNPs and STRs in the *APOA1/C3/A4/A5/BUD13* gene region on Triglycerides

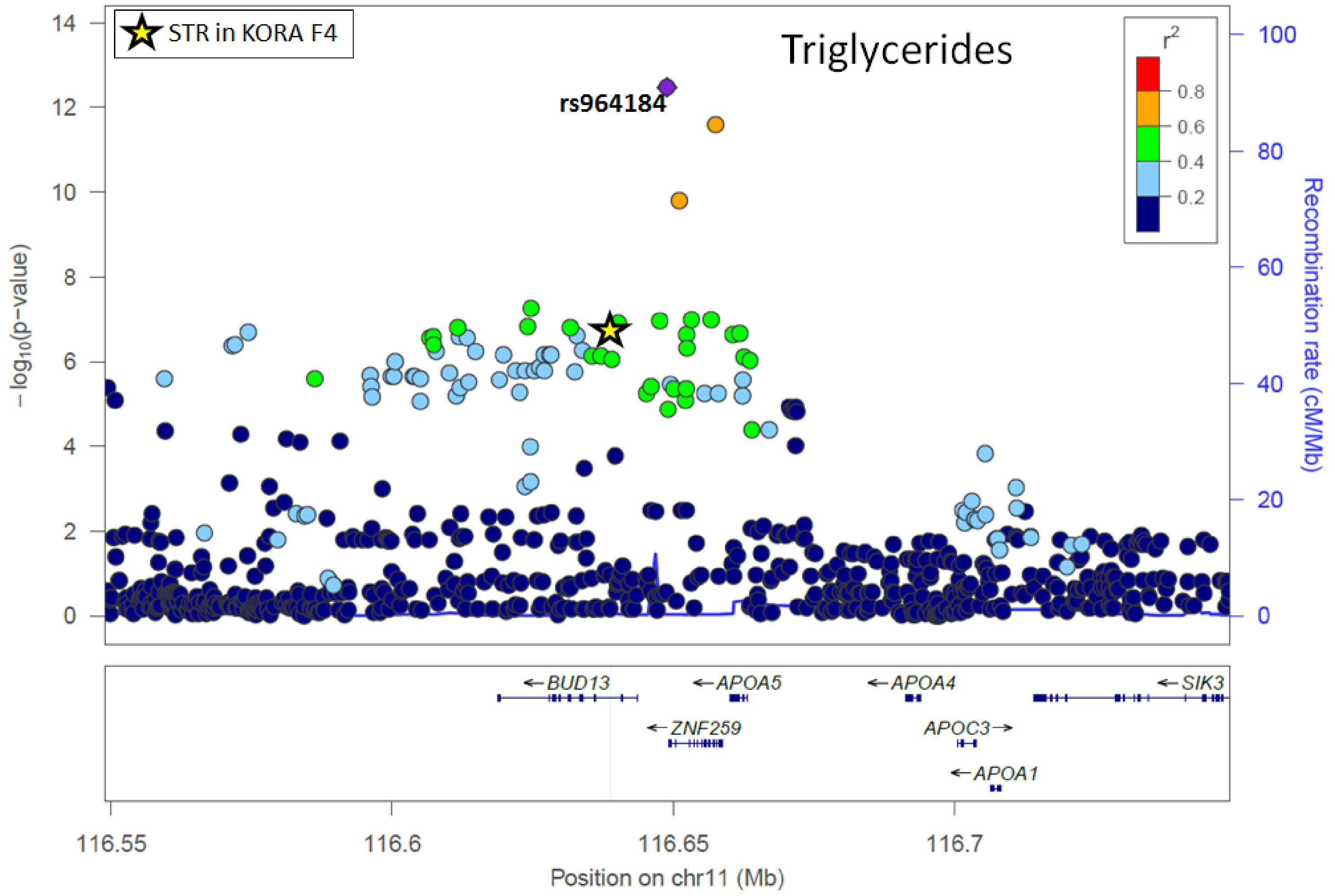

Figure S3k: Regional plots showing the association of SNPs and STRs in the *LPL* gene region on Triglycerides

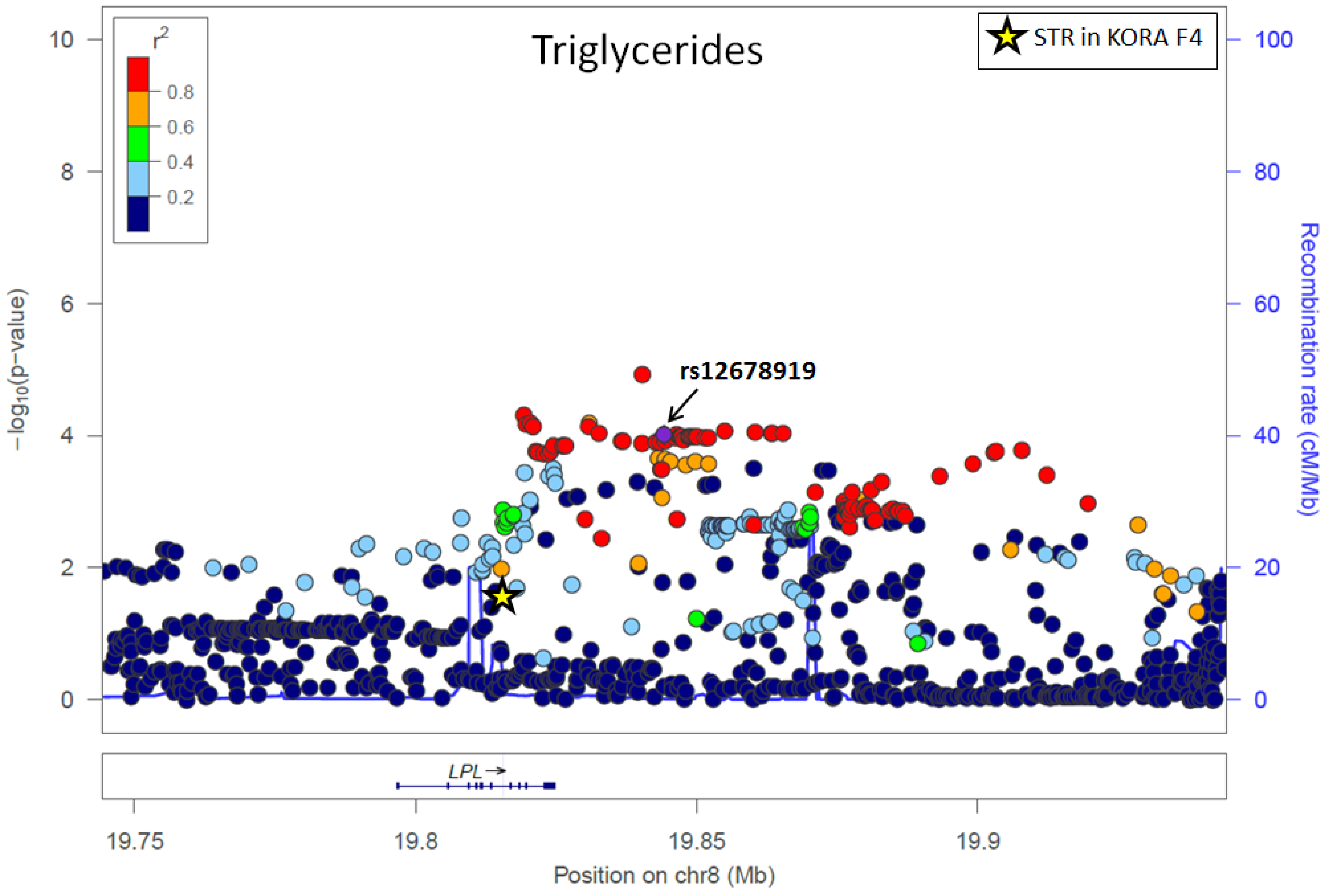

Figure S4a

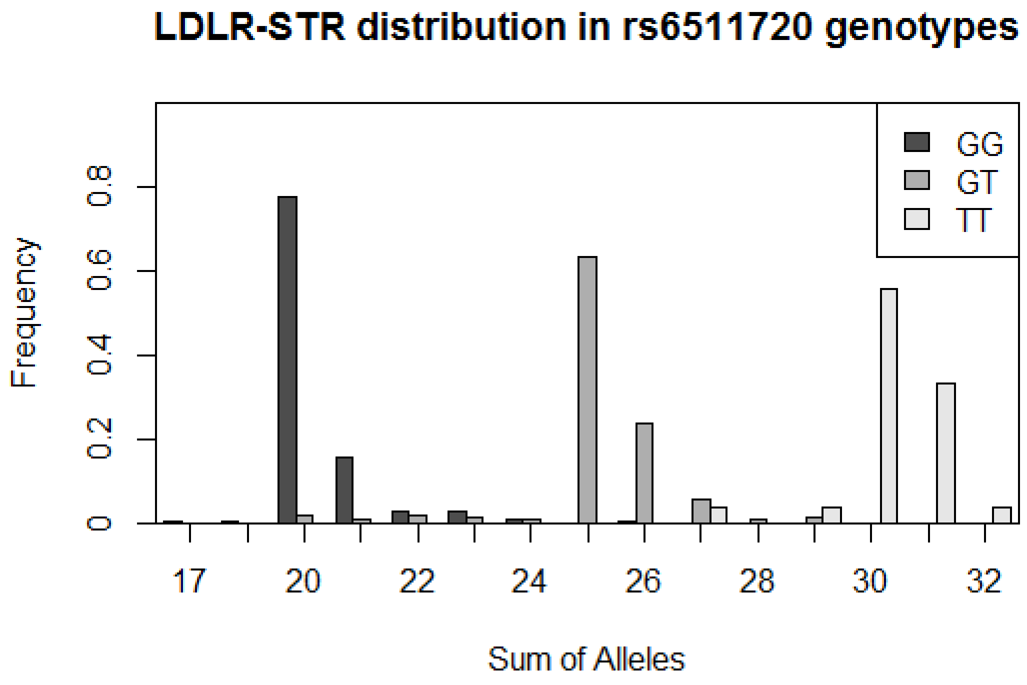

Figure S4b

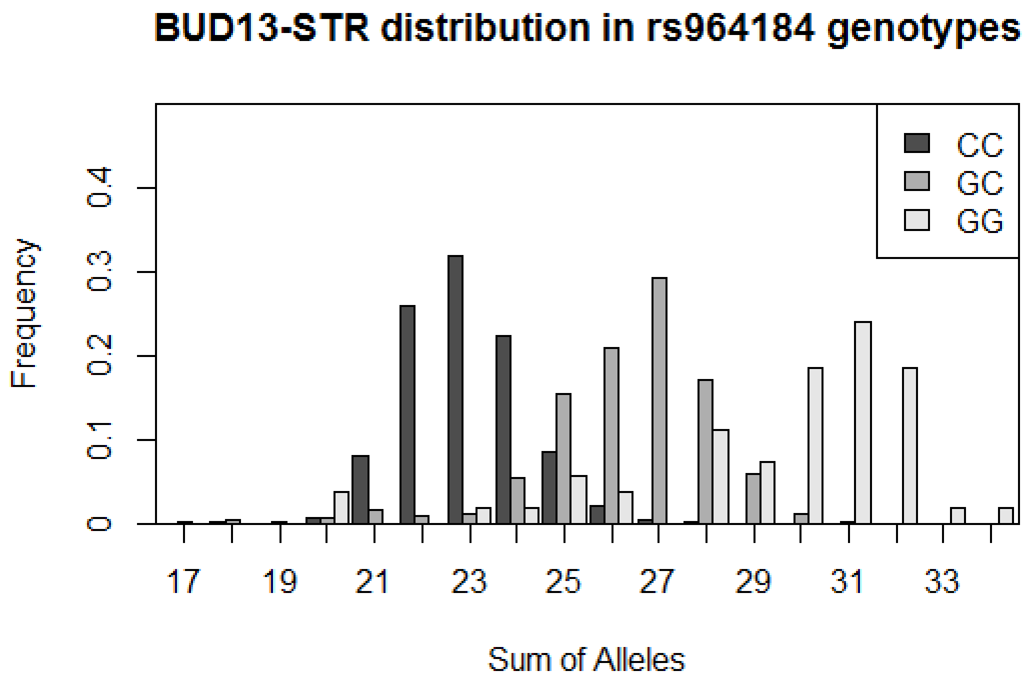

Figure S4c

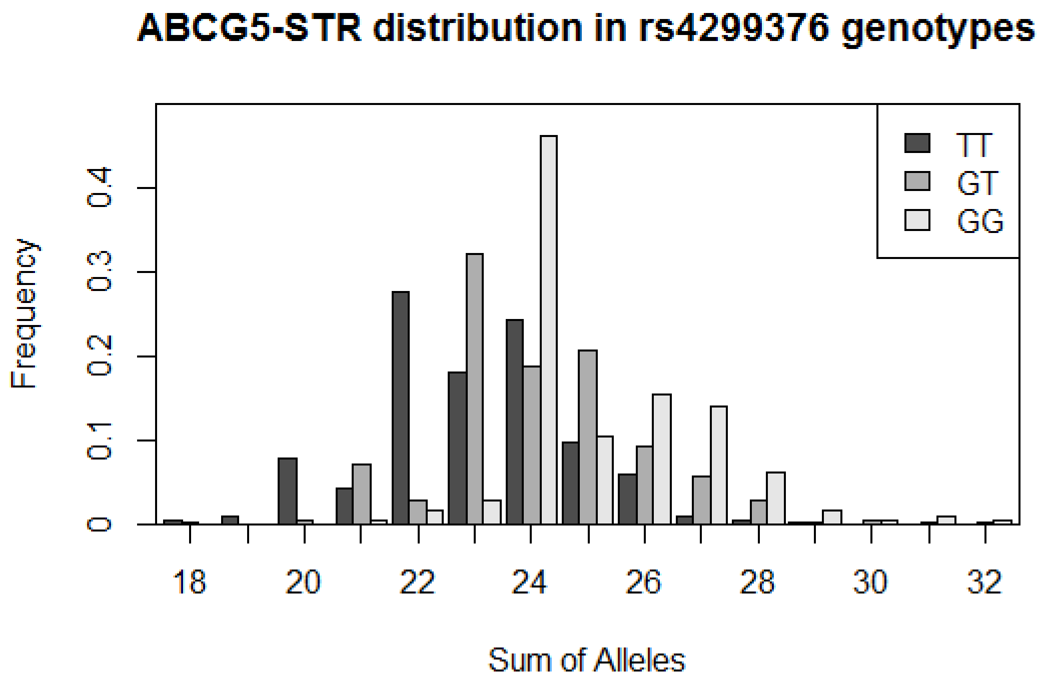

Figure S4d

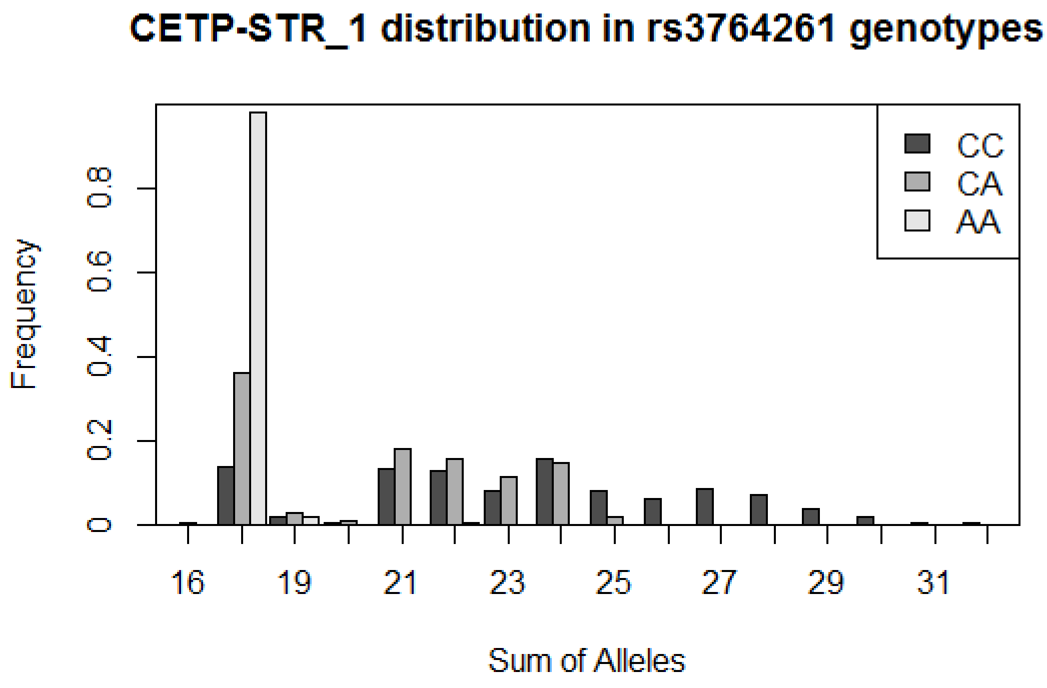

Figure S4e

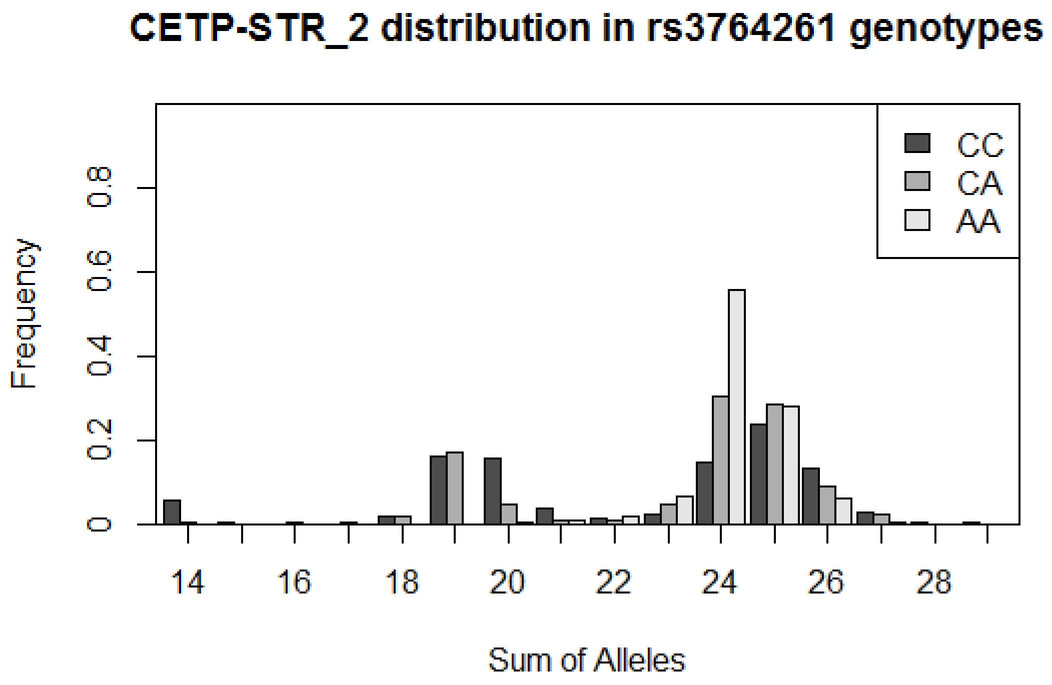

Figure S4f

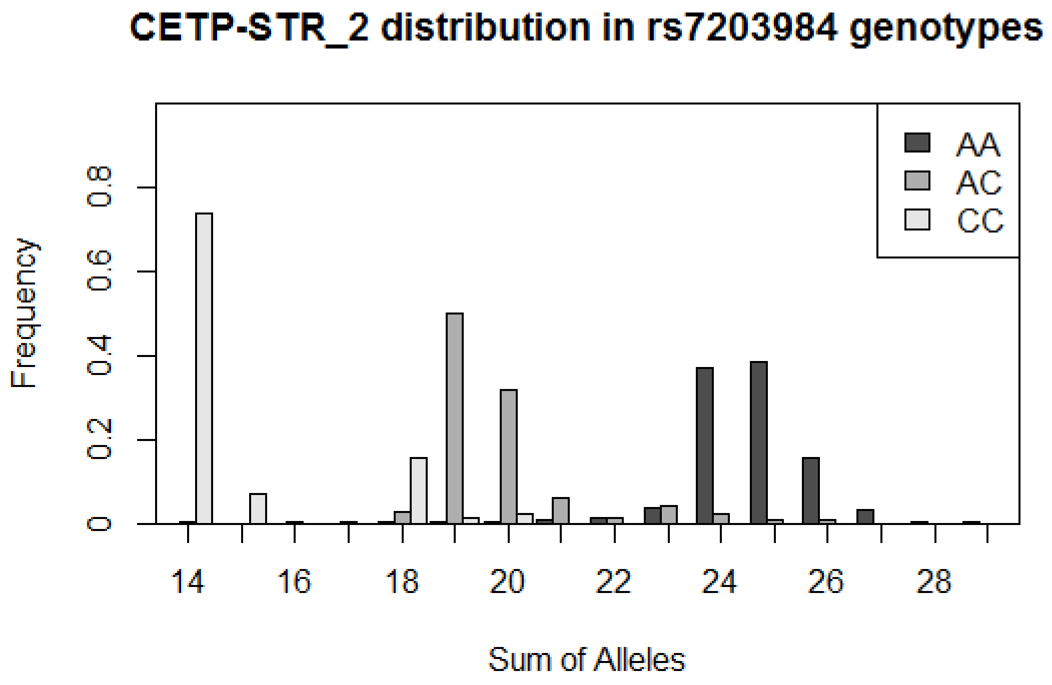

Figure S4g

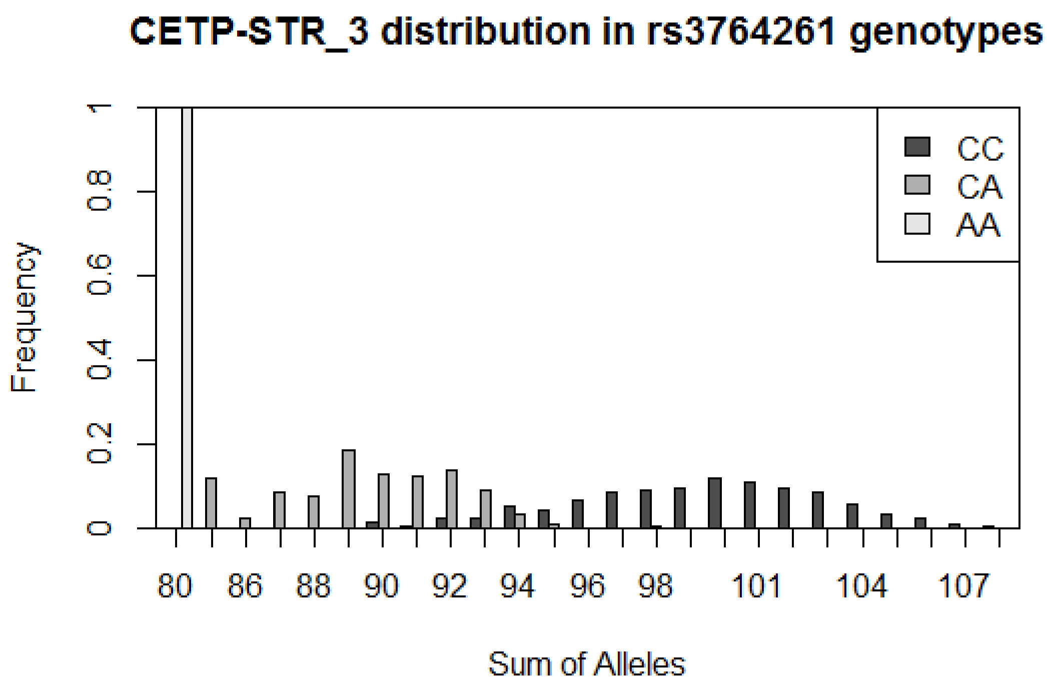

Figure S4h

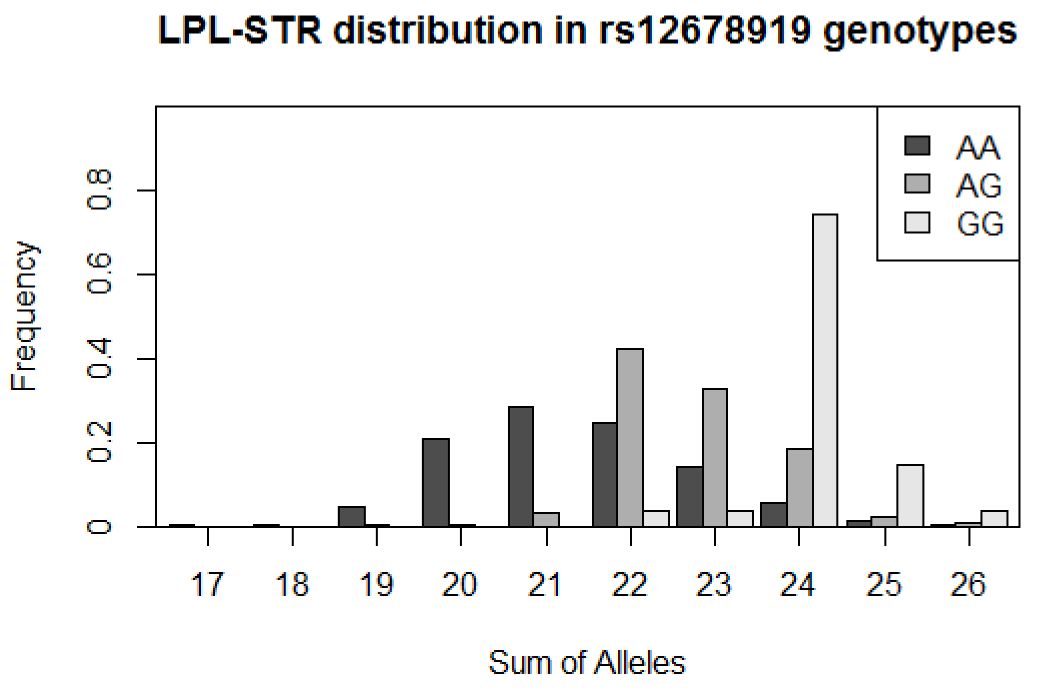

Supplement: File S1 — (PDF) [file pone.0102113.s001.pdf]
